# Lysophosphatidylinositol-Acyltransferase-1 (LPIAT1) Is Required to Maintain Physiological Levels of PtdIns and PtdInsP_2_ in the Mouse

**DOI:** 10.1371/journal.pone.0058425

**Published:** 2013-03-05

**Authors:** Karen E. Anderson, Anna Kielkowska, Tom N. Durrant, Veronique Juvin, Jonathan Clark, Len R. Stephens, Phillip T. Hawkins

**Affiliations:** 1 Inositide Laboratory, Babraham Institute, Babraham Research Campus, Cambridge, United Kingdom; 2 Babraham Bioscience Technologies Ltd., Babraham Research Campus, Babraham, Cambridge, United Kingdom; UAE University, United Arab Emirates

## Abstract

We disrupted the gene encoding lysophosphatidylinositol-acyltransferase-1 (LPIAT1) in the mouse with the aim of understanding its role in determining cellular phosphoinositide content. LPIAT1^−/−^ mice were born at lower than Mendelian ratios and exhibited a severe developmental brain defect. We compared the phospholipid content of livers and brains from LPIAT1^−/−^ and LPIAT1^+/+^ littermates by LC-ESI/MS. In accord with previous studies, the most abundant molecular species of each phosphoinositide class (PtdIns, PtdInsP, PtdInsP_2_ and PtdInsP_3_) possessed a C38∶4 complement of fatty-acyl esters (C18∶0 and C20∶4 are usually assigned to the *sn*-1 and *sn*-2 positions, respectively). LPIAT1^−/−^ liver and brain contained relatively less of the C38∶4 species of PtdIns, PtdInsP and PtdInsP_2_ (dropping from 95–97% to 75–85% of the total species measured for each lipid class) and relatively more of the less abundant species (PtdInsP_3_ less abundant species were below our quantification levels). The increases in the less abundant PtdIns and PtdInsP_2_ species did not compensate for the loss in C38∶4 species, resulting in a 26–44% reduction in total PtdIns and PtdInsP_2_ levels in both brain and liver. LPIAT1^−/−^ brain and liver also contained increased levels of C18∶0 lyso-PtdIns (300% and 525% respectively) indicating a defect in the reacylation of this molecule. LPIAT1^−/−^ brain additionally contained significantly reduced C38∶4 PC and PE levels (by 47% and 55% respectively), possibly contributing to the phenotype in this organ. The levels of all other molecular species of PC, PE, PS and PA measured in the brain and liver were very similar between LPIAT1^−/−^ and LPIAT1^+/+^ samples. These results suggest LPIAT1 activity plays a non-redundant role in maintaining physiological levels of PtdIns within an active deacylation/reacylation cycle in mouse tissues. They also suggest that this pathway must act in concert with other, as yet unidentified, mechanisms to achieve the enrichment observed in C38∶4 molecular species of phosphoinositides.

## Introduction

Individual classes of membrane phospholipids are known to comprise families of molecular species that differ in their fatty acyl composition [1], [2]. Within a lipid class, the individual functions of these different molecular species are poorly understood, but fatty acyl chains which differ in chain length and degree of saturation are generally assumed to convey different biophysical properties to their parent lipid (e.g. unsaturated fatty acyl chains increase mobility in a bilayer) [1] or to act as a reservoir for potential molecular signals (e.g. the release of arachidonic acid through PLA_2_-catalysed hydrolysis) [3]. Phospholipids are synthesised de novo from common DAG or PtdOH precursors (the ‘Kennedy pathway’) and current evidence suggests this fatty acyl diversity is created through subsequent head group-specific deacylation/reacylation pathways (the ‘Lands cycle’) [2].

PtdIns species are unusual amongst major classes of phospholipid in that they are often found as a highly restricted distribution of molecular species in any particular organism, for example ≥85% of PtdIns species in primary mammalian cells or tissues have been measured as the C38∶4 species, with an C18∶0 (stearoyl) chain in the *sn*-1 position and a C20∶4 (arachidonoyl) chain in the *sn-*2 position [4]–[6]. The specific properties of C38∶4 PtdIns that are important in mammalian cells are unknown but are unlikely to reside simply in being a potential source of free C20∶4, since PLA_2_ activities appear to be directed against several phospholipids with higher absolute amounts of C20∶4 [6]. PtdIns is converted through a series of kinase and phosphatase-catalysed reactions into a series of polyphosphorylated species (PtdIns3P, PtdIns4P, PtdIns5P, PtdIns(3,5)P_2_, PtdIns(3,4)P_2_, PtdIns(4,5)P_2_ and PtdIns(3,4,5)P_3_) that are collectively termed phosphoinositides. Most of these polyphosphorylated species have variously well understood functions as molecular signals which regulate membrane events in different cellular locations e.g. trafficking through the endosomal/lysosomal system, trafficking through the Golgi or delivering proximal cell-surface receptor signalling at the plasma membrane [7]. There is very little data which has measured the fatty acyl complement of the more highly phosphorylated phosphoinositides but the available evidence suggests they are very similar to that of the PtdIns from which they were directly or indirectly synthesised [4], [8]–[10], and thus it is probable that the specific fatty acyl composition of phosphoinositides is related to one or more of their signalling functions.

Recent studies have identified a lysophosphatidylinositol-acyltransferase (LPIAT1; [11]) in worms (Mboa-7; [12]), mammals (MBOAT7; [13]) and flies (Farjavit; [14]) with remarkable specificity for lysoPtdIns and an unsaturated-CoA (C20∶4 in mammals and flies). Further, disrupting the expression of the orthologue of this enzyme in C. elegans suggests it is a major determinant of *sn*-2 acyl specificity in PtdIns in this organism [12] and, moreover, that this specificity is important for phosphoinositide function [15]. We disrupted the orthologue of this gene in the mouse (*Lpiat1*) to investigate a role for this enzyme in dictating the fatty acyl complement of phosphoinositides in mammals. We found that loss of LPIAT1 resulted in severe defects in brain development and present an analysis of its impact on the fatty acyl content of the major classes of phospholipids in the brain and liver of these animals. Our results indicate that LPIAT1 plays a major role in sustaining synthesis of C38∶4 phosphoinositides in the brain and liver, and C38∶4 PE and PC in the brain.

## Materials and Methods

### Ethics Statement

All work was submitted to and approved by the Animal Welfare Ethics Committee at the Babraham Institute under Home Office Project license PPL 80/2335.

### Materials

Internal standards 1-heptadecanoyl-2-hexadecanoyl-sn-glycero-3-(phosphoinositol-3,4,5-trisphosphate) (C17∶0/C16∶0-PtdIns(3,4,5)P_3_, as a hepta-sodium salt), C17∶0/C16∶0-PtdIns(4,5)P_2_, C17∶0/C16∶0-PtdIns, C17∶0/C16∶0-phosphatidylserine (PS) and C17∶0/C16∶0-phosphatidic acid (PA) were synthesized at the Babraham Institute. Synthetic 17∶0-lysoPA, 17∶1-lysoPtdIns, C17∶0/C20∶4-phosphatidylcholine (PC) and C17∶0/C20∶4-phosphatidylethanolamine (PE) internal standards were from Avanti Polar lipids. All chemicals/solutions were AR grade.

### Generation of LPIAT1 Knockout Mice

**Figure 1:**
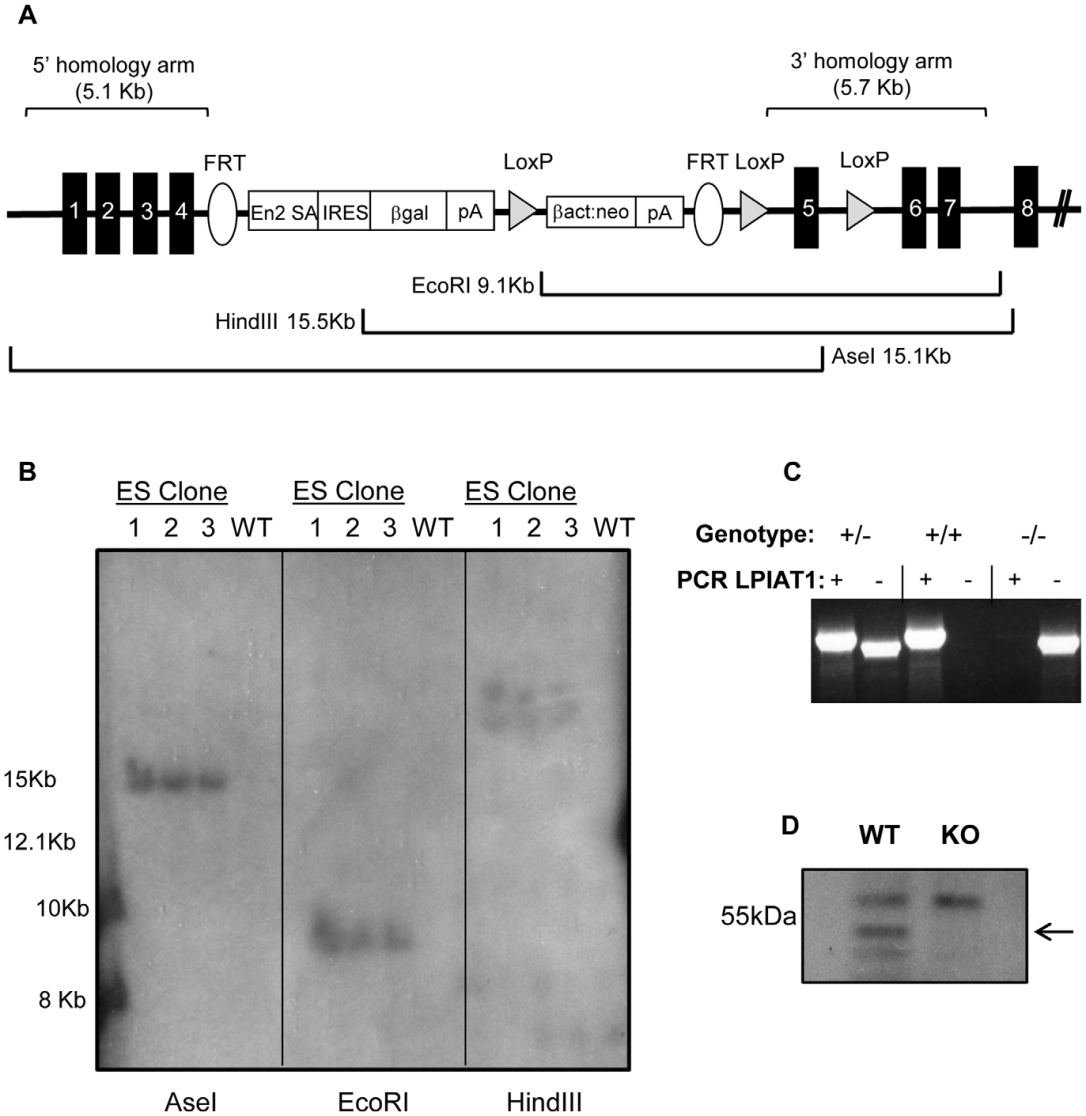
Generation of LPIAT1 knockout first mice. (**A**) Schematic representation of LPIAT^tm1a(KOMP)Wtsi^ gene targeting vector, constructed by the High throughput gene targeting group at the Sanger Center as a ‘knock-out first’ allele that abrogates expression of the targeted allele. Shown are expected enzymatic digest fragments for EcoRI, HindIII and AseI digests of the correctly targeted allele, which were confirmed by Southern analysis of three ES cell clones and WT BL6 genomic DNA control using a [^32^P]-oligonucleotide probe to a 600bp region of the Neomycin gene within the targeting cassette (**B**). (**C**) Genotyping of mice was performed by PCR amplification to identify the presence of LPIAT1 WT allele (+) and/or targeted KO allele (−) from LPIAT1^+/−^, LPIAT1^+/+^ and LPIAT1^−/−^ mice. (**D**) Western blot analysis was performed on 50 µg wet weight brain tissue from three pairs of LPIAT1^+/+^ (WT) and LPIAT1^−/−^ (KO) 13 day old littermates as described in Materials and Methods. Shown is a representative immunoblot, with the arrow indicating the position of LPIAT1 protein.

LPIAT1/MBOAT7 knock-out mice were generated using targeted JM8.N4 ES cells (MBOAT7^tm1a(KOMP)Wtsi^ ) created by the High Throughput Gene Targeting group at the Wellcome Trust Sanger Institute, UK. Correct targeting of the LPIAT1 locus was confirmed in house by Southern blotting using an internal [^32^P]-oligonucleotide probe generated to a 600bp region of the neomycin gene within the targeting cassette, following digest of ES cell-derived genomic DNA with the restriction endonucleases EcoRI, HindIII and AseI (see Figure 1). One correctly-targeted ES cell clone (EPD0094_4_C11) was injected into C57/Bl6Tyr^−/−^-derived blastocysts by the Gene Targeting Facility at the Babraham Institute and male chimeras were mated with C57/Bl6Tyr^−/−^ females. Mice were housed in the Biological Services Unit at the Babraham Institute under specific pathogen-free conditions.

Germline transmission was confirmed and genotyping of mice was routinely performed by PCR amplifications, with detection of the non-targeted WT allele between exon 4 and exon 6 (forward primer 5′ GGGCCTGGGGCTCACCTTATTCACCTGTGGC, reverse primer 5′ CCAGTGGGAAGAGATGGGAGGAC) and/or targeted knockout (KO) allele between exon 4 and lacZ within the targeting cassette (forward primer as for WT, reverse primer 5′ GGATGTGCTGCAAGGCGATTAAGTTGG).

### Tissue Harvesting

Liver and brain tissue was harvested from 13 day old LPIAT1^+/+^ (WT) and LPIAT1^−/−^ (KO) littermates, rinsed rapidly in ice-cold PBS and immediately frozen in liquid N_2_. Samples were ground to a fine powder under liquid N_2_, and stored at −80°C until use.

### Lipid Analysis

5–10 mg wet weight of finely ground liver or brain tissue from WT and KO mice (equivalent to 350–700 and 200–400 µg protein for liver and brain respectively) prepared as described above were resuspended in primary extraction solution (CHCl_3_/MeOH/1M HCl (484/242/23.22): H_2_O 750∶170 v/v) to a concentration of 5 mg tissue per 920 µl and solubilized by probe sonication. For neutral loss scans 5 mg of tissue was analyzed (see Figure S1). For all targeted multiple reaction monitoring (MRM) analysis, unless otherwise indicated (see Figure S2), samples were diluted 1/10 in primary extraction mix for analysis of 0.5 mg wet tissue. For these targeted MRM analyses an internal standard mix (C16:C17-PA (10 ng), C16:C17-PS (100 ng), C16:C17-PtdIns (100 ng), C16:C17-PtdInsP_3_/C16:C17-PtdInsP_2_ (10 ng), C17∶0-lyosPA (50 ng), C17∶1-lysoPtdIns (50 ng), C17∶0/C20∶4-PC (50 ng) and C17∶0/C20∶4-PE (50 ng)) was added to initial extract resuspensions. Lipids were extracted, derivatized with trimethylsilyl-diazomethane, and PtdInsP_3_, PtdInsP_2_ and PS analyzed by mass spectrometry, employing neutral loss of derivatized head groups, using a ABSciex QTRAP4000 connected to a Waters Acquity UPLC system as described in Clark *et al.* [4]. Lyso-phospholipids, PC, PE and PA were also analyzed by this method with appropriate modifications made to the targetted MRM transitions. Verifications of these modified MRM transitions for lyso-PE/PC/PA/PS/PtdIns and PC/PE/PA were performed using relevant synthetic standards, prior to sample analysis. MRM transition parameters for all targeted lipids are shown in Data S1. Protein determinations on triplicate samples of each brain and liver tissue were performed by Bradford assay, with samples prepared as described in Clark *et al*. [4].

Data are shown as nmol/mg protein, calculated by normalizing the MRM targeted lipid integrated response area to that of a known amount of relevant internal standard (ng lipid). Where no direct internal standard was available, responses were normalized as follows: lyso-PC and lyso-PE responses were normalized to PC and PE internal standards respectively, while PtdInsP responses were normalized to the C16:C17-PtdInsP_2_ internal standard. Data were then converted to nmol based on the molecular weight of the relevant lipid species (ng/nmole). Data were analyzed by t-test, with Dunn-Sidak correction for multiple comparisons.

### Western Blotting

60 µg (wet weight) ground brain tissue from both WT and targeted KO mice, solubilized in SDS-sample buffer by bath sonication, were analyzed. Samples were subjected to SDS-PAGE, transferred and immunoblotted for LPIAT1 using 5.0 µg/ml rabbit polyclonal antibody (anti-LENG4 AV49811 Sigma), in TBS/5% milk/0.05% Tween, and HRP-conjugated goat anti-rabbit secondary (sc-2054 Santa Cruz). Signals were detected by ECL+ (GE Healthcare).

## Results

The gene encoding LPIAT1 was disrupted in mouse JM8.N4 ES cells using a gene targeting vector containing a strong polyadenylation consensus signal (Figure 1A). Correct targeting was confirmed by Southern Analysis (Figure 1B). Blastocyst injection and breeding of the resultant chimeras with C57/Bl6Tyr^−/−^ WT mice generated LPIAT1^+/−^ progeny that were, in turn, interbred to create LPIAT1^+/+^ (WT), LPIAT1^+/−^ (HET), LPIAT1^−/−^ (KO) littermates, initially identified by genotyping (Figure 1C). Such breeding revealed significantly fewer live LPIAT^−/−^ births than predicted by Mendelian frequencies (Table 1). LPIAT1^−/−^ mice were significantly smaller than their littermates (54±9.5% of age- and sex-matched WTs) and exhibited a characteristically deformed, domed-shaped head (Figure S1), in addition to an unsteady gait, piloerection, lethargy and difficulties in feeding. This phenotype was very similar to the brain development phenotype reported recently by Vogel *et al.* [16] and Lee *et al.* [5]. LPIAT1^−/−^ mice did not survive for more than 2–4 weeks. Western blot analysis with anti-LPIAT1 antibody confirmed that LPIAT1^−/−^ mice expressed no detectable LPIAT1 protein (Figure 1D).

**Table 1:** Numbers of genotypes in litters from LPIAT1^+/−^ crosses.

	LPIAT1^+/+^ (WT)	LPIAT1^+/−^ (HET)	LPIAT1^−/−^ (KO)
No. live births	55	99	17
% of total	32.2	57.9	9.9

Tissues were collected from LPIAT1^+/+^ and LPIAT1^−/−^ littermates at 13 days of age and their phospholipid content was analyzed by LC-ESI/MS. The main focus of our study was to investigate the impact of LPIAT1 deletion on phosphoinositides. We therefore used an approach we have described recently which can accurately quantify phosphoinositide molecular species in small amounts of tissue [4]. This approach uses standard lipid extraction in acidified solvents followed by derivatization with TMS-diazomethane. TMS-diazomethane was used to efficiently esterify the phosphate groups of the phosphoinositides, allowing more efficient ionisation and detection of these species in the mass spectrometer. Derivatized extracts were then separated by LC on a C4 column before analysis by ESI^+^ MS using MRM transitions defined using appropriate synthetic standards (see methods). Initial studies indicated several major phospholipid species could be measured accurately in these derivatized extracts using this approach.

**Figure 2:**
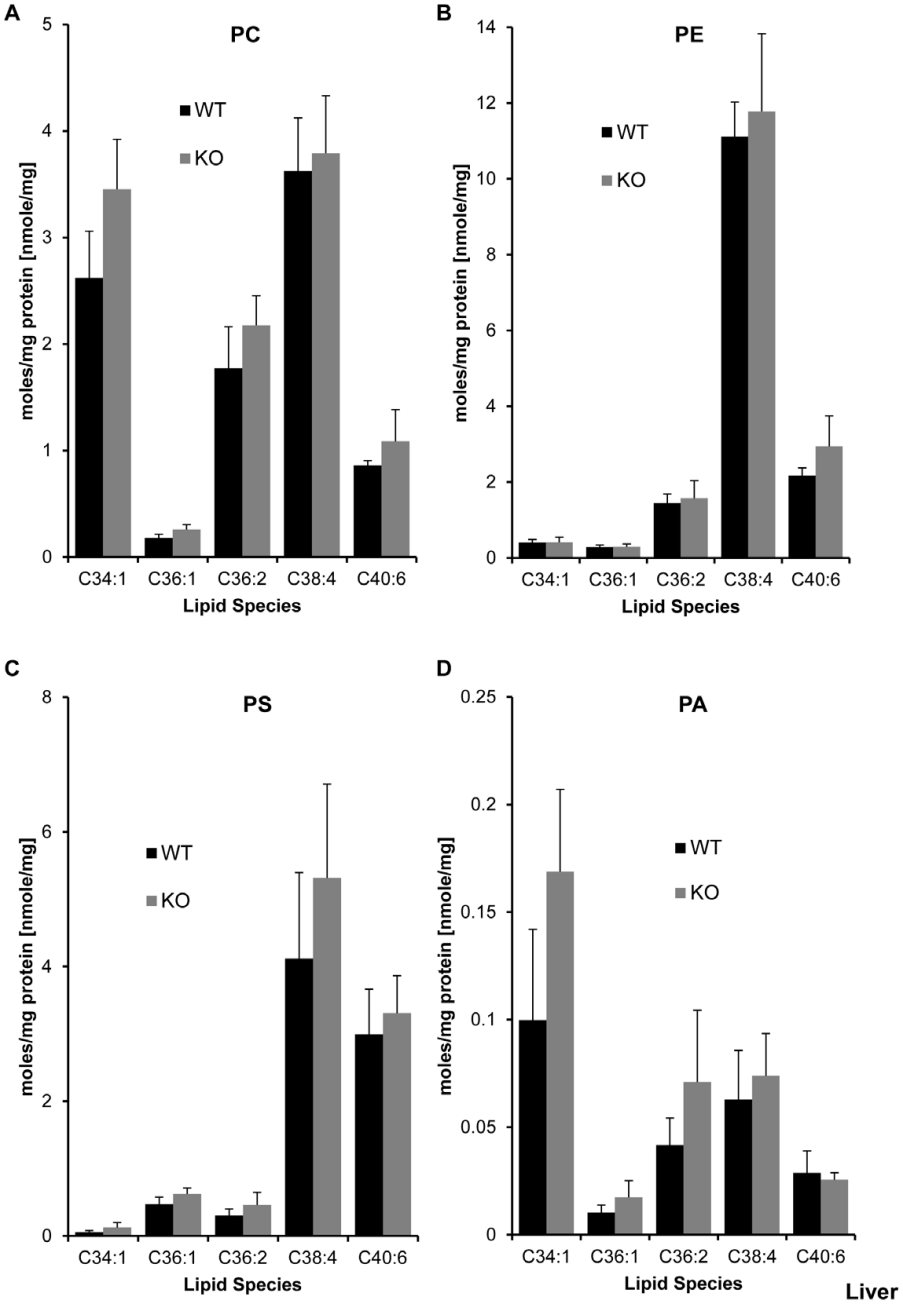
Effect of LPIAT1 knockout on liver phospholipid molecular species. Livers from 13 day old littermates expressing (LPIAT1^+/+^ (WT)) or lacking (LPIAT1^−/−^ (KO)) LPIAT1 were homogenized and lipids extracted from 0.5 mg wet weight as described in Materials and Methods. Targeted molecular species of PC (**A**), PE (**B**), PS (**C**) and PA (**D**) were detected by MRM mass spectrometric analysis as described in Materials and Methods. Data are expressed as moles/mg protein, normalized to relevant internal standards. Shown are mean ± SD, n = 4 for both WT and KO. Data were analyzed by T-test.

**Figure 3:**
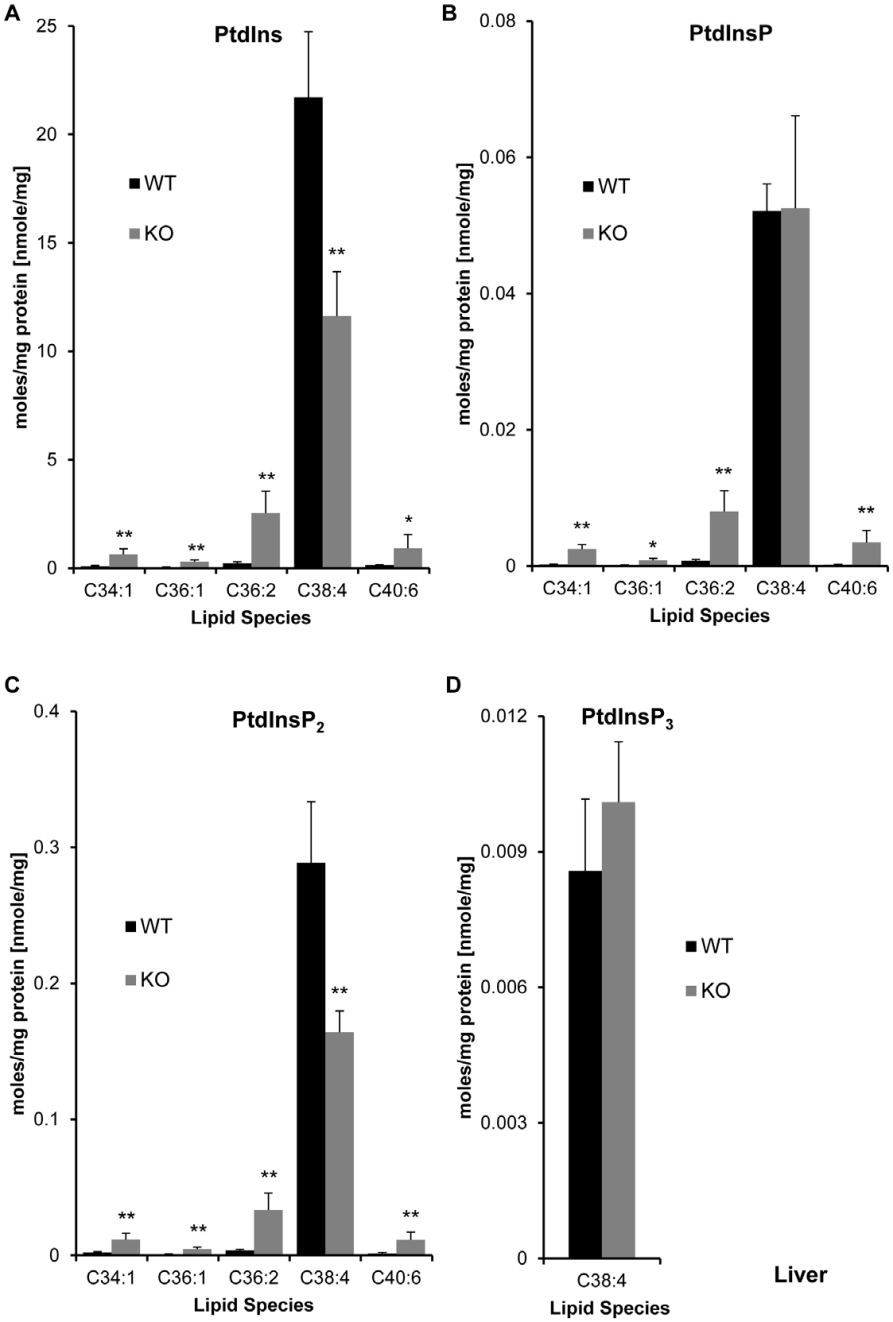
Effect of LPIAT1 knockout on liver phosphoinositide lipid molecular species. Livers from 13 day old littermates expressing (LPIAT1^+/+^ (WT)) or lacking (LPIAT1^−/−^ (KO)) LPIAT1 were homogenized and lipids extracted from 0.5 mg wet weight as described in Materials and Methods. Targeted molecular species of PtdIns (**A**), PtdInsP (**B**), PtdInsP_2_ (**C**) and PtdInsP_3_ (**D**) were detected by MRM mass spectrometric analysis as described in Materials and Methods. Data are expressed as moles/mg protein, normalized to relevant internal standards. Shown are mean ± SD, n = 4 for both WT and KO. Data were analyzed by T-test. *p≤0.05, **p≤0.005.

**Figure 4:**
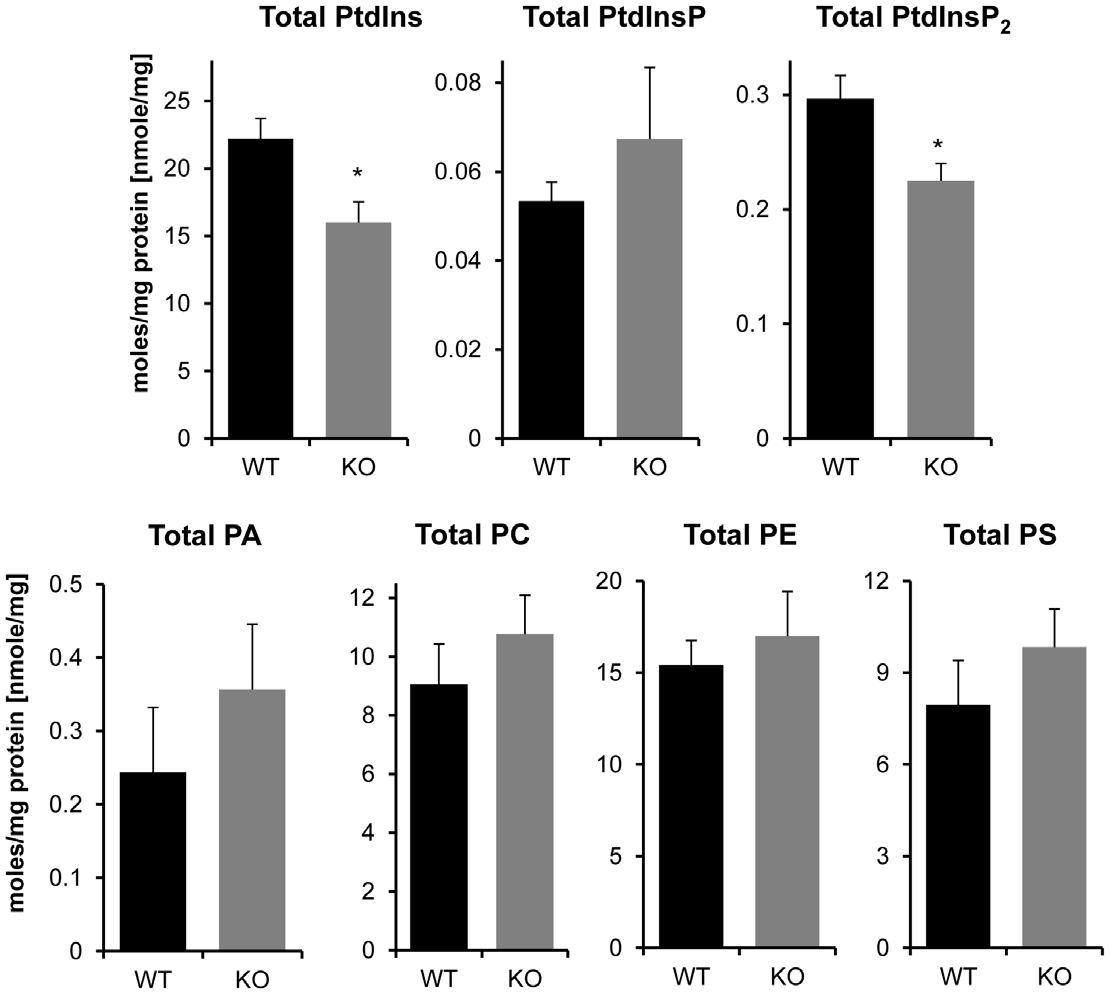
Effect of LPIAT1 knockout on total levels of liver phospholipids and phosphoinositides. Quantitated targeted molecular species of phosphoinositides PtdIns, PtdInsP and PtdInsP_2_ in LPIAT1^+/+^ (WT) or LPIAT1^−/−^ (KO) liver samples presented in Figure 3, and phospholipids PA,PC,PE and PS from Figure 2 were added to calculate total levels for each lipid. Data are mean ± SD, n = 4 for both WT and KO. *p≤0.05, T-test.

**Figure 5:**
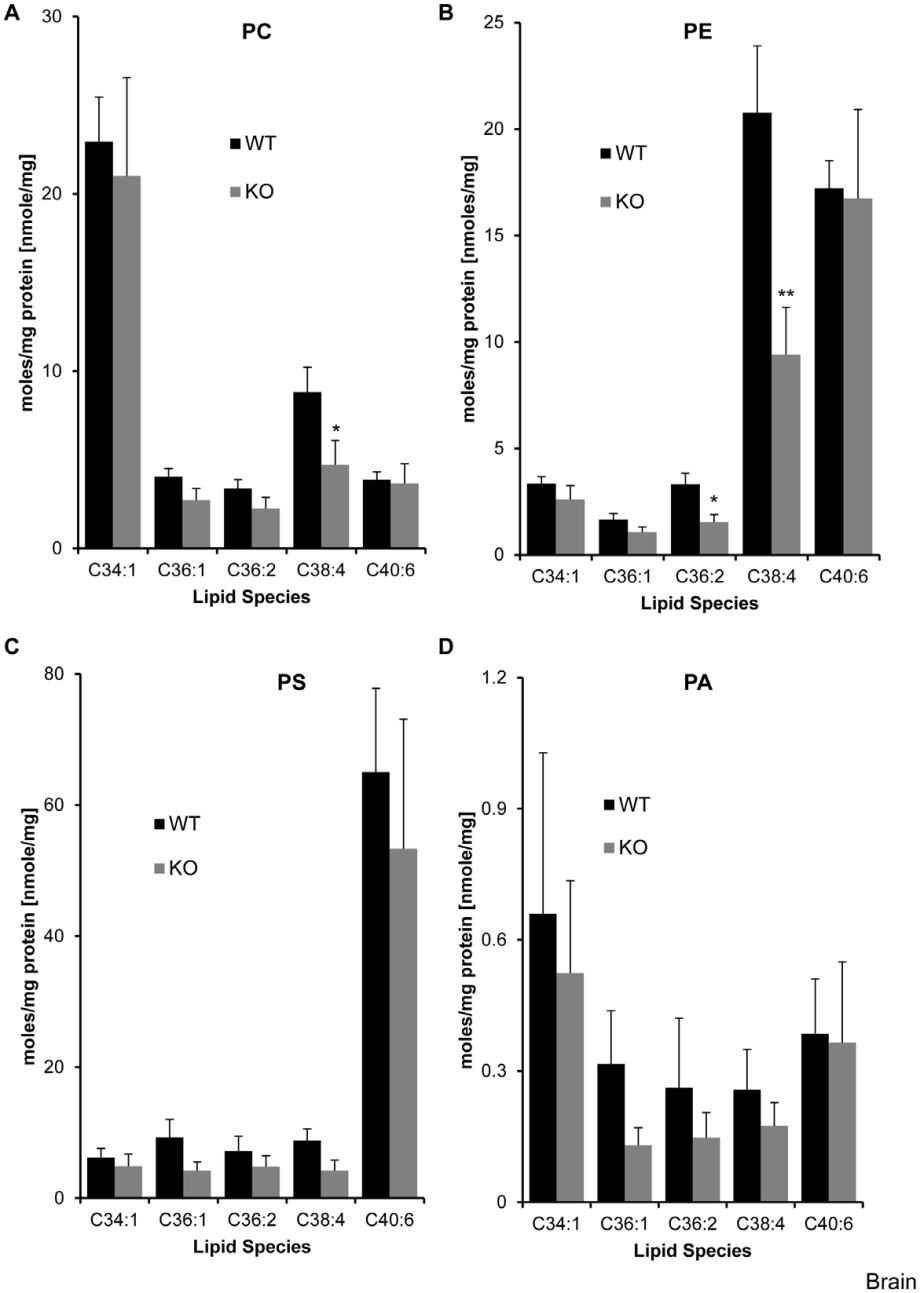
Effect of LPIAT1 knockout on brain phospholipid molecular species. Brains from 13 day old littermates expressing (LPIAT1^+/+^ (WT)) or lacking (LPIAT1^−/−^ (KO)) LPIAT1 were homogenized and lipids extracted from 0.5 mg wet weight as described in Materials and Methods. Targeted molecular species of PC (**A**), PE (**B**), PS (**C**) and PA (**D**) were detected by MRM mass spectrometric analysis as described in Materials and Methods. Data are expressed as moles/mg protein, normalized to relevant internal standards. Shown are mean ± SD, n = 4 for both WT and KO. Data were analyzed by T-test. *p≤0.05, **p≤0.005.

**Figure 6:**
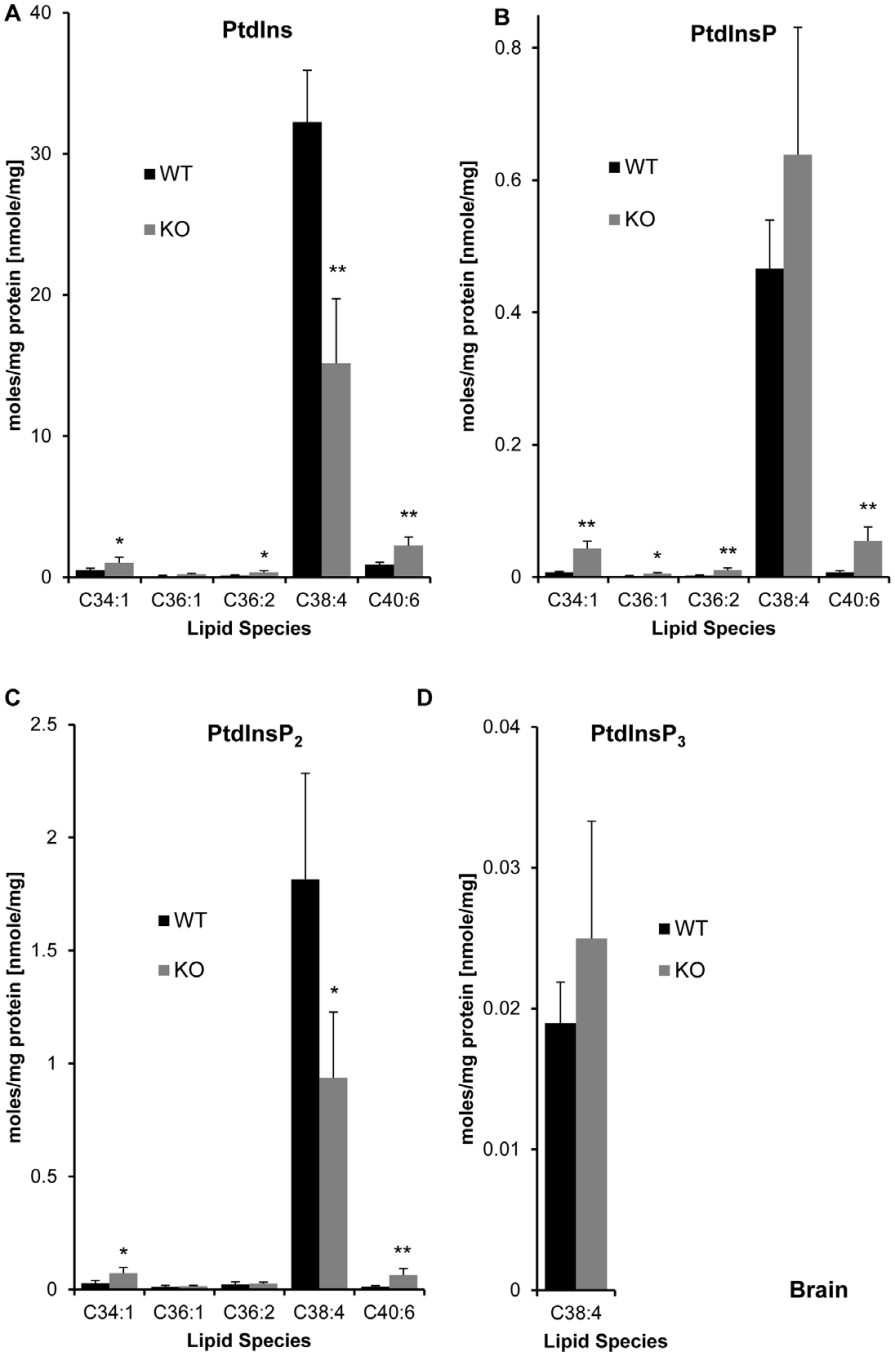
Effect of LPIAT1 knockout on brain phosphoinositide lipid molecular species. Brains from 13 day old littermates expressing (LPIAT1^+/+^ (WT)) or lacking (LPIAT1^−/−^ (KO)) LPIAT1 were homogenised and lipid extracted from 0.5 mg wet weight as described in Materials and Methods. Targeted molecular species of PtdIns (**A**), PtdInsP (**B**), PtdInsP_2_ (**C**) and PtdInsP_3_ (**D**) were detected by MRM mass spectrometric analysis as described in Materials and Methods. Data are expressed as moles/mg protein, normalized to relevant internal standards. Shown are mean ± SD, n = 4 for both WT and KO. Data were analyzed by T-test. *p≤0.05, **p≤0.005.

**Figure 7:**
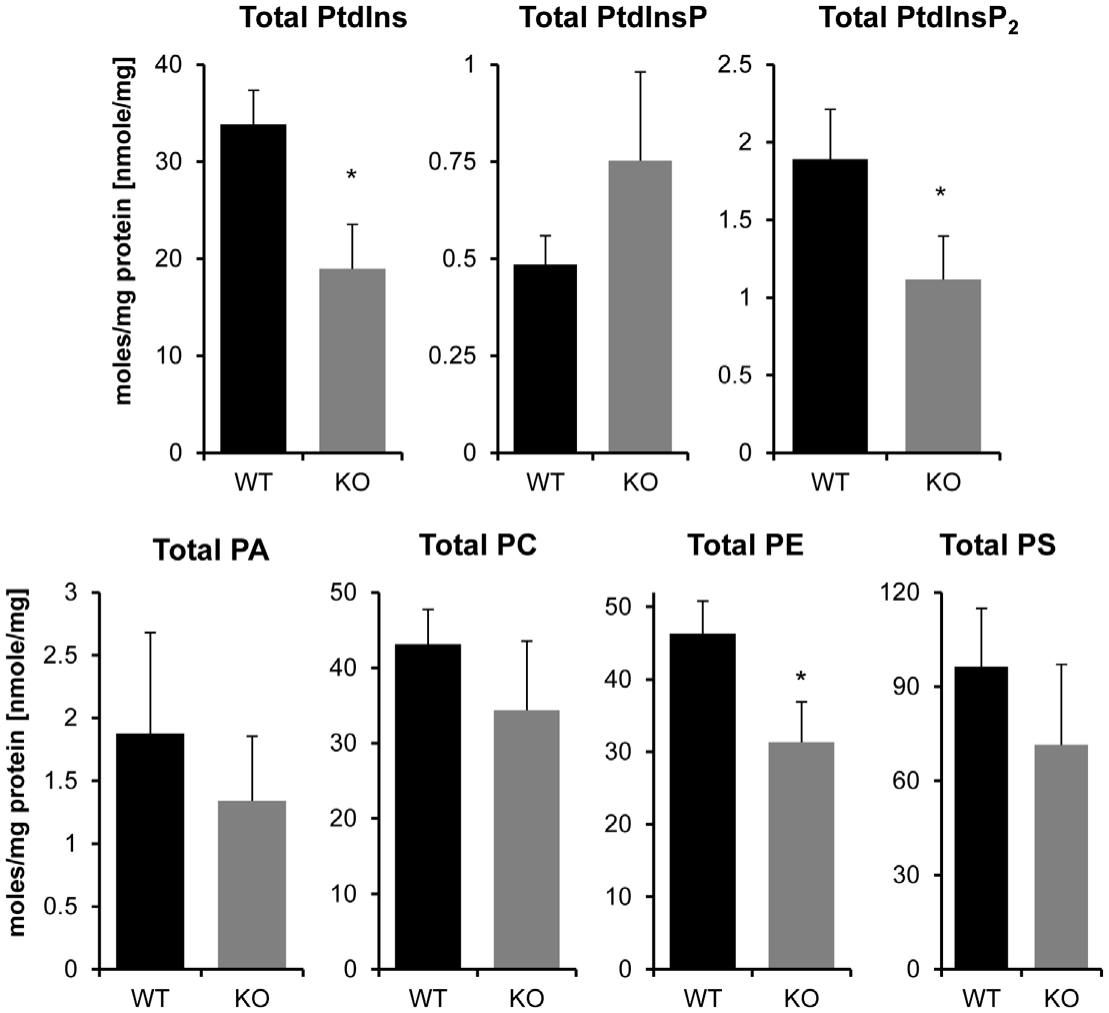
Effect of LPIAT1 knockout on total levels of brain phospholipids and phosphoinositides. Quantitated targetted molecular species of phosphoinositides PtdIns, PtdInsP and PtdInsP_2_ in LPIAT1^+/+^ (WT) or LPIAT1^−/−^ (KO) brain samples presented in Figure 6, and phospholipids PA,PC,PE and PS from Figure 5 were added to calculate total levels for each lipid. Data are mean ± SD, n = 4 for both WT and KO. *p≤0.05, **p≤0.005, T-test.

**Figure 8:**
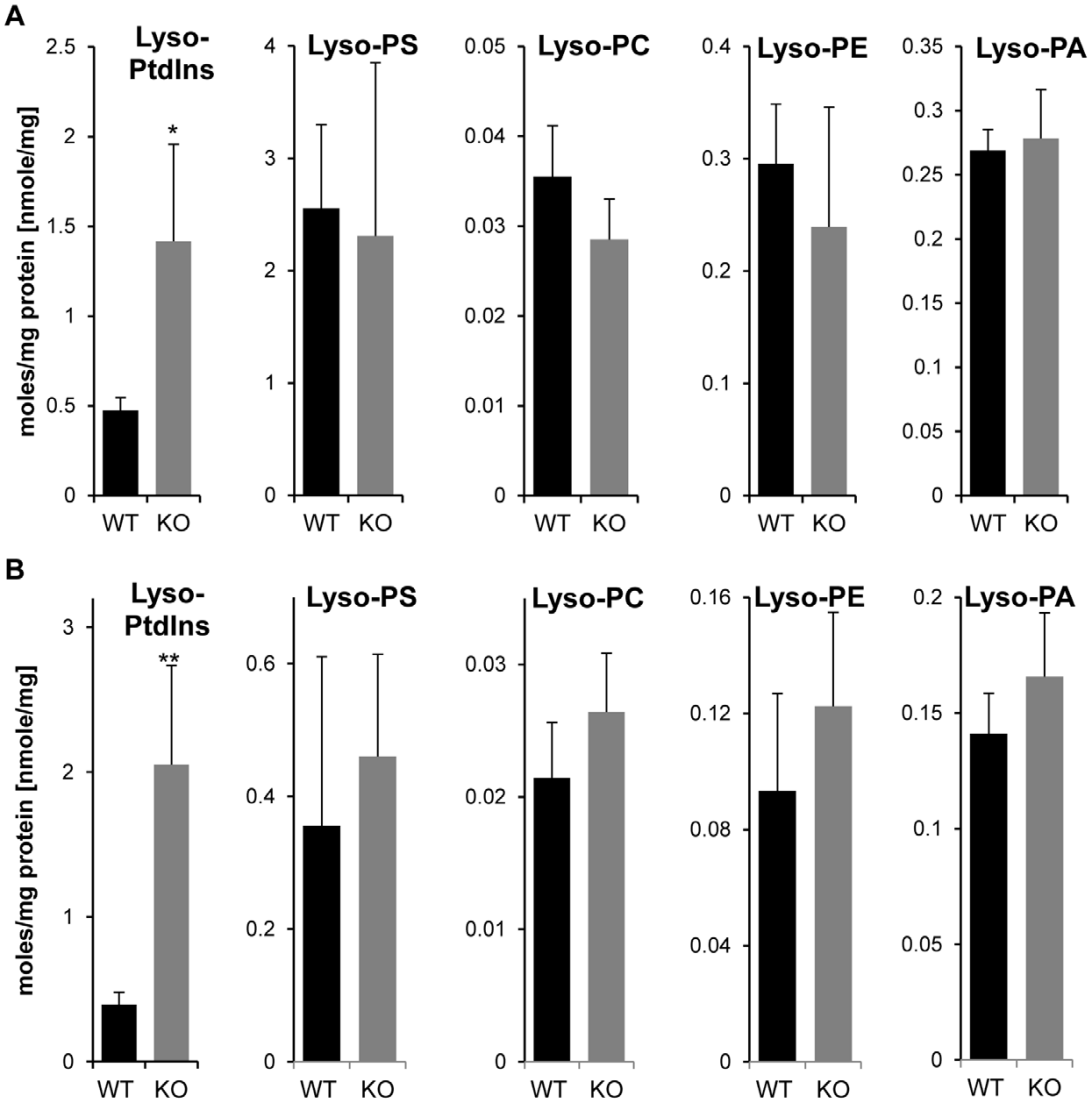
Effect of LPIAT1 knockout on brain and liver lyso-phospholipid molecular species. Brains (**A**) or livers (**B**) from 13 day old littermates expressing (LPIAT1^+/+^ (WT)) or lacking (LPIAT1^−/−^ (KO)) LPIAT1 were ground, homogenized and lipids extracted from 0.5 mg wet weight as described in Materials and Methods. Lyso-phospholipids were targeted and were detected by MRM mass spectrometric anaylsis as described in Materials and Methods. Data are expressed as moles/mg protein, normalized to relevant internal standards. Shown are mean ± SD, n = 4 for both WT and KO. Data were analyzed by T-test. *p≤0.05, **p≤0.005.

We first identified the most abundant molecular species present in several tissues (brain, liver, spleen, heart, kidney, skeletal muscle, lung) for both the major phospholipid classes (PC,PS,PE,PA) and the phosphoinositides (PtdIns,PtdInsP,PtdInsP_2_,PtdInsP_3_), by measuring DAG^+^ species created through the neutral loss in mass of the derivatized head-group (‘neutral loss scans’). Neutral loss scans are presented for the brain and liver in Figures S2, S3, S4, S5. On the basis of these scans, five molecular species for each lipid class were chosen for further quantitation. These individual molecular species were quantified by integrating appropriate MRM measurements in the LC eluate and correcting them for recovery using a closely matched internal standard (for a particular lipid class, these were usually a synthetic standard with a C17∶0 in the sn-1 position and an appropriate unsaturated fatty acid in the *sn*-2 position; see Methods). Initial experiments indicated this approach yielded linear measurements for each of these lipid species in a tissue range of 0.25 to 2 mg wet weight (Figure S6). The data presented in Figures 2, 3, 4, 5, 6, 7, 8 are presented as nmol/mg protein derived from a 0.5 mg wet weight sample of frozen liver or brain (equivalent to approximately 35 µg and 20 µg protein for liver and brain samples respectively).

Loss of LPIAT1 had remarkably little effect on the levels of each species of PC, PE, PS and PA measured in liver extracts, with no significant differences observed in any of the lipid species (Figure 2, Figure S7) or total levels of each lipid (Figure 4) between WT and KO samples. In contrast, loss of LPIAT1 did have a significant impact on the levels of phosphoinositide species (Figure 3). As anticipated, the major species of PtdIns measured was C38∶4 (greater than 95% of total species measured, see Figure S8). Further, the spread in relative abundance of C38∶4 and the four other, minor species (C34∶1, C36∶1, C36∶2 and C40∶6) were very similar between PtdIns, PtdInsP and PtdInsP_2_, reflecting their accepted biosynthetic route of sequential phosphorylation. PtdInsP_3_ species were present at such low levels that only the C38∶4 species could be quantified accurately. Loss of LPIAT1 reduced the levels of C38∶4 PtdIns and PtdInsP_2_ by 46 and 43% respectively, but had no significant effect on the levels of C38∶4 PtdInsP and C38∶4 PtdInsP_3_ (Figure 3). Loss of LPIAT1 in the liver also significantly raised the levels of the minor species of PtdIns, PtdInsP and PtdInsP_2_ (Figure 3). However, these increases in the minor species of PtdIns and PtdInsP_2_ did not compensate for the loss of C38∶4 PtdIns and PtdInsP_2_, such that loss of LPIAT1 resulted in an approximate 30% drop in total levels of PtdIns and PtdInsP_2_ in the liver (Figure 4).

An equivalent analysis is presented for the abundance of the relevant lipid species in total brain extracts (Figures 5, 6, 7). PC, PE, PS and PA species showed substantially different relative distributions in the brain compared to the liver, as expected from numerous previous studies [17] indicating tissue-specific acylation of phospholipids [18]. Loss of LPIAT1, in marked contrast to liver extracts, caused a clear and specific reduction in C38∶4 species of PE and PC by approximately 50% in LPIAT1^−/−^ brains (see also Figure S9) which, for PE at least, was reflected in a significant decrease in total brain levels (Figure 7).

As in the liver, the dominant species of phosphoinositides in the brain was C38:4 (Figure S10). Further, loss of LPIAT1 caused very similar changes in brain phosphoinositides to those seen in the liver, with a 53% and 48% reduction in the levels of C38:4 PtdIns and PtdInsP_2_, respectively, but the levels of C38:4 PtdInsP and PtdInsP_3_ remained unchanged. There was also a clear increase in the levels of the minor species of each phosphoinositide in LPIAT1^−/−^ extracts, though again they did not compensate for the reduction in levels of the C38:4 species (Figure 7), with total levels of PtdIns and PtdInsP_2_ dropping by 44% and 42%, respectively.

The very significant drop in PtdIns levels in LPIAT1^−/−^ brain and liver suggested LPIAT activity is required to maintain PtdIns synthesis in the context of significant PLA_2_ activity. To investigate this we also measured the levels of the relevant lysophospholipid species in brain and liver. Loss of LPIAT1 specifically and significantly elevated the levels of C18∶0 lysoPtdIns in both brain and liver extracts (Figure 8), though these accumulations were substantially less than the loss of C38∶4 PtdIns.

## Discussion

During the course of this study two other groups independently created mice lacking expression of LPIAT1 and described a severe defect in brain development, with reduced cerebral cortex and hippocampus found in forebrains from LPIAT1^−/−^ E18.5 embryos [5] manifesting to severe hydrocephalus in the knockout mouse, affecting the lateral and third ventricles [16]. Our observations of the phenotype of the LPIAT1^−/−^ mice we created are entirely in accord with the more detailed descriptions presented in these studies. Lee *et al.* also presented some characterisation of the relative changes in phospholipid species in LPIAT1^−/−^ brains [5]. There are several similarities in the data presented in this later study with the data presented here. In both studies, loss of LPIAT1 resulted in a relative drop in the levels of C38∶4 phosphoinositide species and a concomitant increase in the relative levels of non-C38∶4 species. Lee *et al.* ascribed the phenotype of LPIAT1^−/−^ brains to a relative loss of C20∶4 in the *sn*-2 position of phosphoinositides, however they did not present quantification of the absolute levels of any species. Our data suggest a more complex interpretation. We describe several changes in the molecular species of lipids in LPIAT1^−/−^ brains that may drive the developmental defect; a decrease in C38∶4 PtdIns or PtdInsP_2_, an increase in lyso-PtdIns or a decrease in C38∶4 PE and PC. Moreover, it is not possible at present to deduce whether the C38∶4 species of PtdIns or PtdInsP_2_ have a specific role, because LPIAT1^−/−^ mice do not compensate adequately for loss of lysoPtdIns acyl transferase activity by re-acylating with other fatty acyl-CoAs i.e. total levels of PtdIns and PtdInsP_2_ levels are not maintained. The less severe phenotypes exhibited by FADS1^−/−^ [19] and FADS2^−/−^ [20], [21] mice (the two desaturases responsible for de novo conversion of C18∶2 to C20∶4) are consistent with the more severe LPIAT1^−/−^ phenotype being due to a drop in the physiological levels of PtdIns and PtdInsP_2_ rather than a change in acyl composition. Although, as pointed out by Lee *et al*, FADS1^−/−^ and FADS2^−/−^ embryos may be protected from early C20∶4 deprivation through accessing this lipid from the mother [5].

The similarities in the effects of loss of LPIAT1 on phosphoinositide levels in brain and liver are striking. In both tissues, loss of LPIAT1 caused a reduction in the proportion of PtdIns, PtdInsP and PtdInsP_2_ that was the C38∶4 species (falling from 95–97% to 75–85% of the total species measured) and an increase in the levels of other, minor species. This re-distribution of molecular species was not seen for any other phospholipid class and suggests LPIAT1 activity using C20∶4-acylCoA normally ‘out competes’ other acyltransferases in the conversion of lysoPtdIns to PtdIns. However, as described above, the approx. 40–50% loss of total PtdIns in LPIAT1^−/−^ brain and liver indicates the activity of these other acyl transferases is insufficient to maintain PtdIns synthesis. The specific rise in C18∶0 lysoPtdIns seen in LPIAT1^−/−^ tissues strongly supports this argument. Taken together, this data indicates that PtdIns undergoes substantial deacylation/re-acylation in both the liver and brain, either as part of a basal lipid re-modelling program (the Lands cycle) or through the regulated release and recapture of C20∶4 (through receptor-controlled pathways designed to synthesise cyclooxygenase/lipoxygenase products); some evidence has been provided for this latter pathway in neutrophils [13]. In either case, our data indicate LPIAT1 activity is required to sustain sufficient reacylation of lyso-PtdIns to maintain PtdIns levels.

Previous studies indicate LPIAT1 cannot use lyso-PtdInsP_2_ as a substrate [5], [22] and therefore the parallel loss of PtdIns and PtdInsP_2_ in LPIAT1^−/−^ brain and liver suggest a drop in PtdIns creates a drop in PtdInsP_2_ through an indirect reduction in its sequential phosphorylation. The lack of any substantial effect of LPIAT1 loss on total PtdInsP levels is therefore confusing, given the accepted abundance of PtdIns(4)P and PtdIns(4,5)P_2_ as the major regio-isomers of PtdInsP and PtdInsP_2_, respectively, and the accepted sequential phosphorylation of PtdIns in the 4- and 5-postions to form PtdIns(4,5)P_2_. Recently, evidence has been provided [23], [24] that the bulk of the PtdInsP pool in mammalian cells is not in dynamic equilibrium with the PtdInsP_2_ pool, therefore it is possible that a small pool of PtdInsP connects PtdIns to PtdInsP_2_ synthesis with a kinetic poise that makes it sensitive to the levels of PtdIns. The lack of any substantial effect of LPIAT1 loss on PtdInsP_3_ levels in brain and liver is more easily explained because it is the terminal product of the phosphorylation cascade, synthesised via 3-phosphorylation of PtdIns(4,5)P_2_. Thus, changes in PtdInsP_3_ might be insulated from changes in PtdInsP_2_ by the catalytic properties of the Class I PI3Ks which catalyse this reaction (e.g. low Km or activation by receptor signalling pathways).

The specific effect of the loss of C38∶4 PE and, to a lesser extent, C38∶4 PC in LPIAT1^−/−^ brain is surprising and suggests LPIAT1 is engaged directly or indirectly in fatty acyl modelling of these lipids in the brain. This may be because LPIAT1 has a wider role relative to other acyltransferases in the brain or, increased deacylation of C20∶4 across PE, PC and PtdIns in the brain places a higher burden on the reacylation capacity of this tissue. It is also possible that major phenotypic changes in brain development in LPIAT1^−/−^ animals create more widespread changes in PE and PC lipids through very indirect mechanisms; though the specificity of the effects for C38∶4 species would argue against this.

Previous studies have indicated the specificity of LPIAT1 for both PtdIns and C20∶4-CoA may underlie the remarkable enrichment of C20∶4 in the *sn*-2 position of phosphoinositides in mammals [5], [12], [13]. Our data independently confirm that 95–97% of phosphoinositides in primary mouse tissues are the C38∶4 species, which comprise C18∶0 and C20∶4 in the *sn*-1 and *sn*-2 positions, respectively [4]–[6] (and data not shown). Careful analysis of the molecular species of phospholipids in LPIAT1^−/−^ tissues indicate LPIAT1 does indeed contribute significantly to the enrichment of C38∶4 species of phosphoinositides but, even in the absence of LPIAT1, approx. 75–85% of phosphoinositide species remain C38∶4 in liver and brain. Thus, although the participation of LPIAT1 in a C20∶4-selective re-acylation cycle may sustain or indeed contribute to C20∶4 enrichment, at least one further mechanism must exist to enrich the *sn-*2 position of these lipids. Either another LPIAT activity is present with specificity for C20∶4-CoA or some selectivity for this fatty acyl species is introduced through the cycle of de novo synthesis and/or recycling of these lipids. Other LPIAT activities have been described but they have not been demonstrated to exhibit a sufficiently compelling specificity to account, in their own right, for the level of C20∶4 enrichment observed [11]. PtdIns is synthesised from CDP-DAG and inositol by PtdIns synthase (PIS), and therefore it is possible that selectivity for C38∶4 species occurs during the cycling of PtdIns through DAG, PA, CDP-DAG and back to PtdIns again. However, although specificity for C38∶4 has been demonstrated for DAG kinase epsilon [25], it is still unclear to what extent this enzyme represents a ‘molecular filter’ for C38∶4 species entering the PtdIns cycle [26]. It is also possible given the recent demonstration that PIS is localised to a discrete, mobile, vesicular pool derived from the ER [27], that specificity for incorporation of fatty acyl chains into PtdIns might be determined by fatty acyl-CoA availability i.e. C20∶4-CoA might be specifically delivered to the PIS-containing organelle. Clearly further studies are needed to address the mechanism by which phosphoinositides are enriched in C18∶0/C20∶4 fatty acyl species and hence provide a route to understanding why evolution has selected this pathway in mammalian cells.

## Supporting Information

Figure S1 **Phenotype of LPIAT1^−/−^ Mice.** Photos of 14 day old mice expressing (LPIAT1^+/+^ (WT)) or lacking (LPIAT1^−/−^ (KO)) LPIAT1, highlighting the decrease in size of KO mice in comparison to their WT littermates, and the display of a domed head.
(TIF)

Figure S2 **Neutral Loss Scans of Phospholipids from derivatized lipid extracts from LPIAT1^+/+^ and LPIAT1^−/−^ liver tissue.** Lipids were extracted from 5 mg of ground liver tissue from LPIAT1^+/+^ (WT) and LPIAT1^−/−^ (KO) mice and analyzed by neutral loss on a QTRAP4000 mass spectrometer as described in Materials and Methods. Shown are neutral loss scans for PC, PE, PS and PA. Labeled are the five lipid molecular species from each scan that were targeted by MRM in subsequent analysis, giving fatty acids from the diacylglycerol unit. ISD = internal standard, cps = counts per second.
(TIF)

Figure S3 **Neutral Loss Scans of Phosphoinositides from derivatized lipid extracts from LPIAT1^+/+^ and LPIAT1^−/−^ liver tissue.** Lipids were extracted from 5 mg of ground liver tissue from LPIAT1^+/+^ (WT) and LPIAT1^−/−^ (KO) mice and analyzed by neutral loss on a QTRAP4000 mass spectrometer as described in Materials and Methods. Shown are neutral loss scans for phosphoinositides PtdIns, PtdInsP and PtdInsP_2_. Labeled are the five lipid molecular species from each scan that were targeted by MRM in subsequent analysis, giving fatty acids from the diacylglycerol unit. cps = counts per second.
(TIF)

Figure S4 **Neutral Loss Scans of Phospholipids from derivatized lipid extracts from LPIAT1^+/+^ and LPIAT1^−/−^ brain tissue.** Lipids were extracted from 5 mg of ground brain tissue from LPIAT1^+/+^ (WT) and LPIAT1^−/−^ (KO) mice and analyzed by neutral loss on a QTRAP4000 mass spectrometer as described in Materials and Methods. Shown are neutral loss scans for PC, PE, PS and PA. Labeled are the five lipid molecular species from each scan that were targeted by MRM in subsequent analysis, giving fatty acids from the diacylglycerol unit. ISD = internal standard, cps = counts per second.
(TIF)

Figure S5 **Neutral Loss Scans of Phosphoinositides from derivatized lipid extracts from LPIAT1^+/+^ and LPIAT1^−/−^ brain tissue.** Lipids were extracted from 5 mg of ground brain tissue from LPIAT1^+/+^ (WT) and LPIAT1^−/−^ (KO) mice and analyzed by neutral loss on a QTRAP4000 mass spectrometer as described in Materials and Methods. Shown are neutral loss scans for phosphoinositides PtdIns, PtdInsP and PtdInsP_2_. Labeled are the five lipid molecular species from each scan that were targeted by MRM in subsequent analysis, giving fatty acids from the diacylglycerol unit. cps = counts per second.
(TIF)

Figure S6 **Linear measurement of phospholipid molecular species with increasing tissue amounts.** Targeted molecular species of PtdIns (**A**), PtdInsP (**B**), PtdInsP_2_ (**C**) and C38∶4 PtdInsP_3_ and PS (**D**) were measured by mass spectrometry from increasing masses (0.25–2 mg tissue, independent dilutions, as indicated) from LPIAT1^+/+^ (WT) or LPIAT1^−/−^ (KO) brain and liver samples, prepared as described in Materials and Methods. Data are presented as response ratios, with measurements normalized to relevant C16:C17-PtdIns, PtdInsP_2_ or PtdInsP_3_ added internal standards. PtdInsP values were normalized to C16:C17-PtdInsP_2_ standard. 0.5 mg samples (highlighted) were used for all subsequent analysis.
(PDF)

Figure S7 **Effect of LPIAT1 knockout on relative amounts of phospholipid molecular species in the liver.** Targeted molecular species of PC (**A**), PE (**B**), PS (**C**) and PA (**D**) from liver samples of mice expressing (LPIAT1^+/+^ (WT)) or lacking (LPIAT1^−/−^ (KO)) LPIAT1 presented in Figure 2 were added to produce total levels of relevant lipids. Each molecular species was then calculated as a percentage of this total lipid value. Shown are mean ± SD, n = 4 for both WT and KO. Data were analyzed by T-test.
(TIF)

Figure S8 **Effect of LPIAT1 knockout on relative amounts of phosphoinositide molecular species in the liver.** Targeted molecular species of PtdIns (**A**), PtdInsP (**B**), and PtdInsP_2_ (**C**) from liver samples of WT or KO mice presented in Figure 3 were added to produce total levels of relevant lipids. Each molecular species was then calculated as a percentage of this total lipid value. Shown are mean ± SD, n = 4 for both WT and KO. Data were analyzed by T-test.
(TIF)

Figure S9 **Effect of LPIAT1 knockout on relative amounts of phospholipid molecular species in the brain.** Targeted molecular species of PC (**A**), PE (**B**), PS (**C**) and PA (**D**) from brain samples of mice expressing (LPIAT1^+/+^ (WT)) or lacking (LPIAT1^−/−^ (KO)) LPIAT1 presented in Figure 5 were added to produce total levels of relevant lipids. Each molecular species was then calculated as a percentage of the total lipid pool. Shown are mean ± SD, n = 4 for both WT and KO. Data were analyzed by T-test.
(TIF)

Figure S10 **Effect of LPIAT1 knockout on relative amounts of phosphoinositide molecular species in the brain.** Targeted molecular species of PtdIns (**A**), PtdInsP (**B**), and PtdInsP_2_ (**C**) from brain samples of WT or KO mice presented in Figure 6 were added to produce total levels of relevant lipids. Each molecular species was then calculated as a percentage of this total lipid value. Shown are mean ± SD, n = 4 for both WT and KO. Data were analyzed by T-test.
(TIF)

Data S1 **MRM Transitions for Analysis of Internal Standards and Tissue Lipids on QTRAP4000 Mass Spectrometer.** MRM transition parameters for detection of internal lipid standards and lipids in brain and liver tissue extracts, showing masses of derivatized head group (Q3- for neutral loss), and corresponding mass of parent lipid species (Q1). Parameters for each lipid were confirmed with synthetic standards.
(PDF)
