# Supplementary figures and images for: Lysophosphatidylinositol-Acyltransferase-1 (LPIAT1) Is Required to Maintain Physiological Levels of PtdIns and PtdInsP2 in the Mouse
Source: PLoS One. 2013 Mar 5;8(3):e58425. doi: 10.1371/journal.pone.0058425 (PMC3589398; doi:10.1371/journal.pone.0058425)

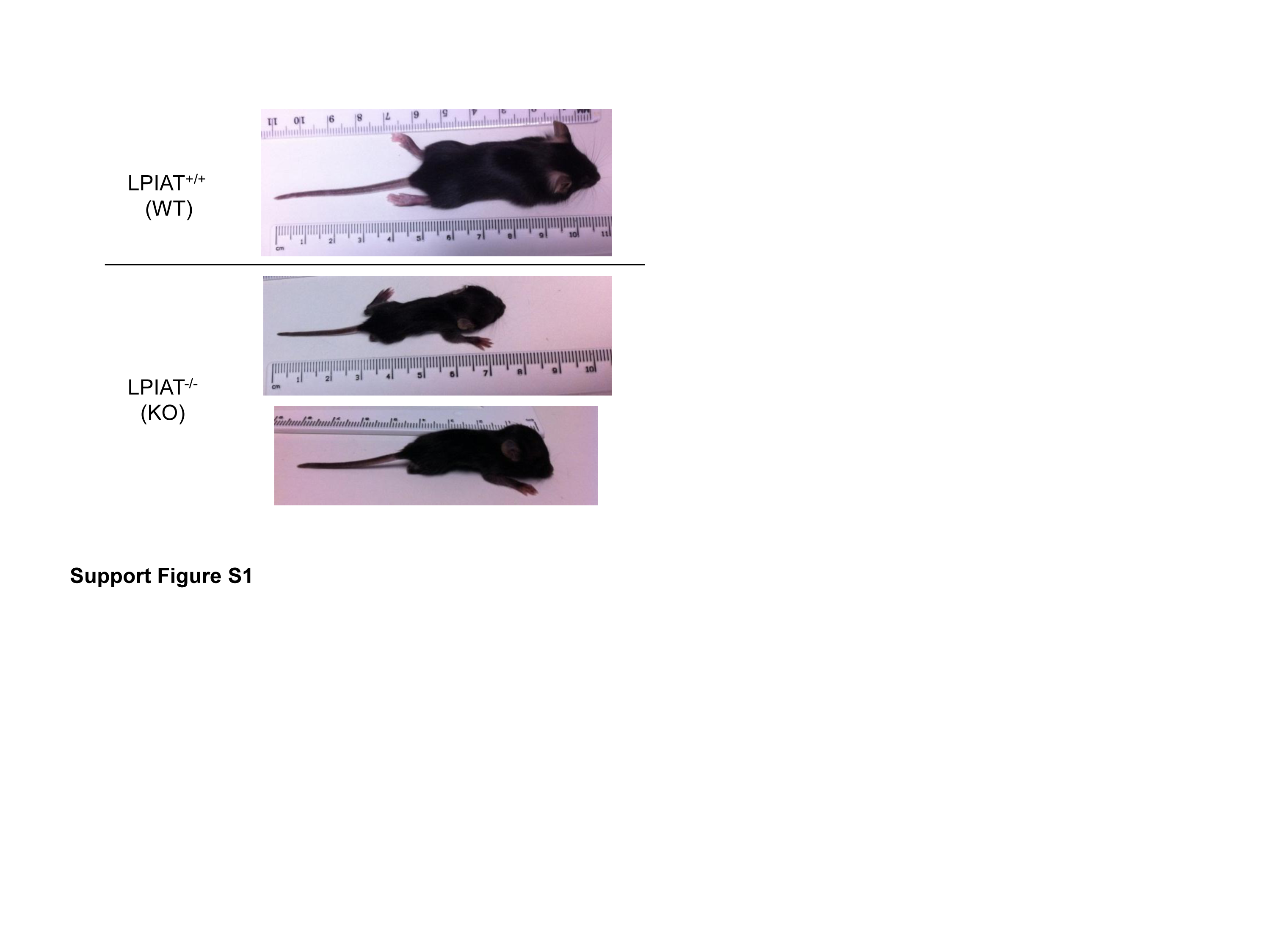

Supplement: Figure S1 — Phenotype of LPIAT1−/− Mice. Photos of 14 day old mice expressing (LPIAT1+/+ (WT)) or lacking (LPIAT1−/− (KO)) LPIAT1, highlighting the decrease in size of KO mice in comparison to their WT littermates, and the display of a domed head. (TIF) [file pone.0058425.s001.tif]

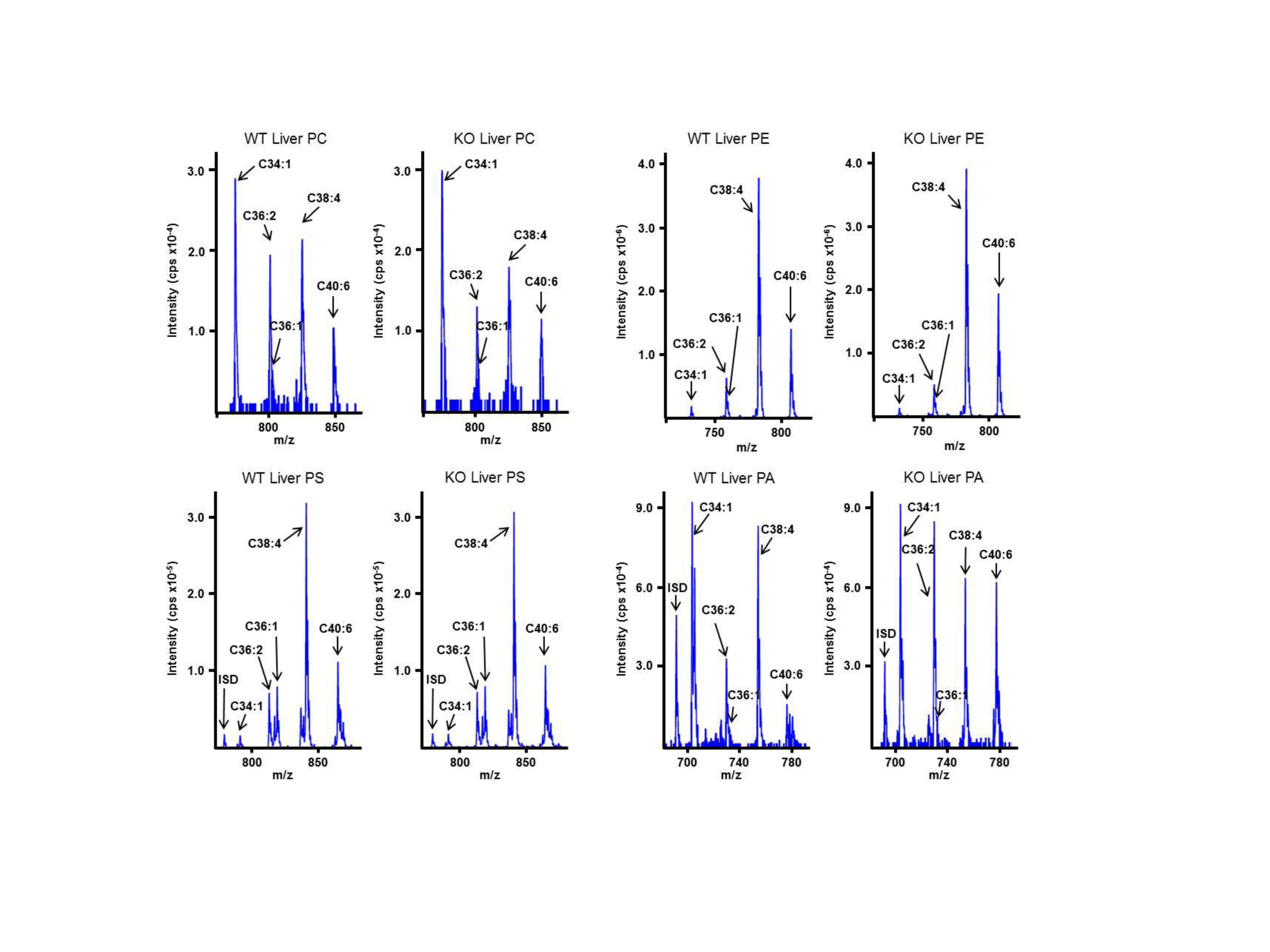

Supplement: Figure S2 — Neutral Loss Scans of Phospholipids from derivatized lipid extracts from LPIAT1+/+ and LPIAT1−/− liver tissue. Lipids were extracted from 5 mg of ground liver tissue from LPIAT1+/+ (WT) and LPIAT1−/− (KO) mice and analyzed by neutral loss on a QTRAP4000 mass spectrometer as described in Materials and Methods. Shown are neutral loss scans for PC, PE, PS and PA. Labeled are the five lipid molecular species from each scan that were targeted by MRM in subsequent analysis, giving fatty acids from the diacylglycerol unit. ISD = internal standard, cps = counts per second. (TIF) [file pone.0058425.s002.tif]

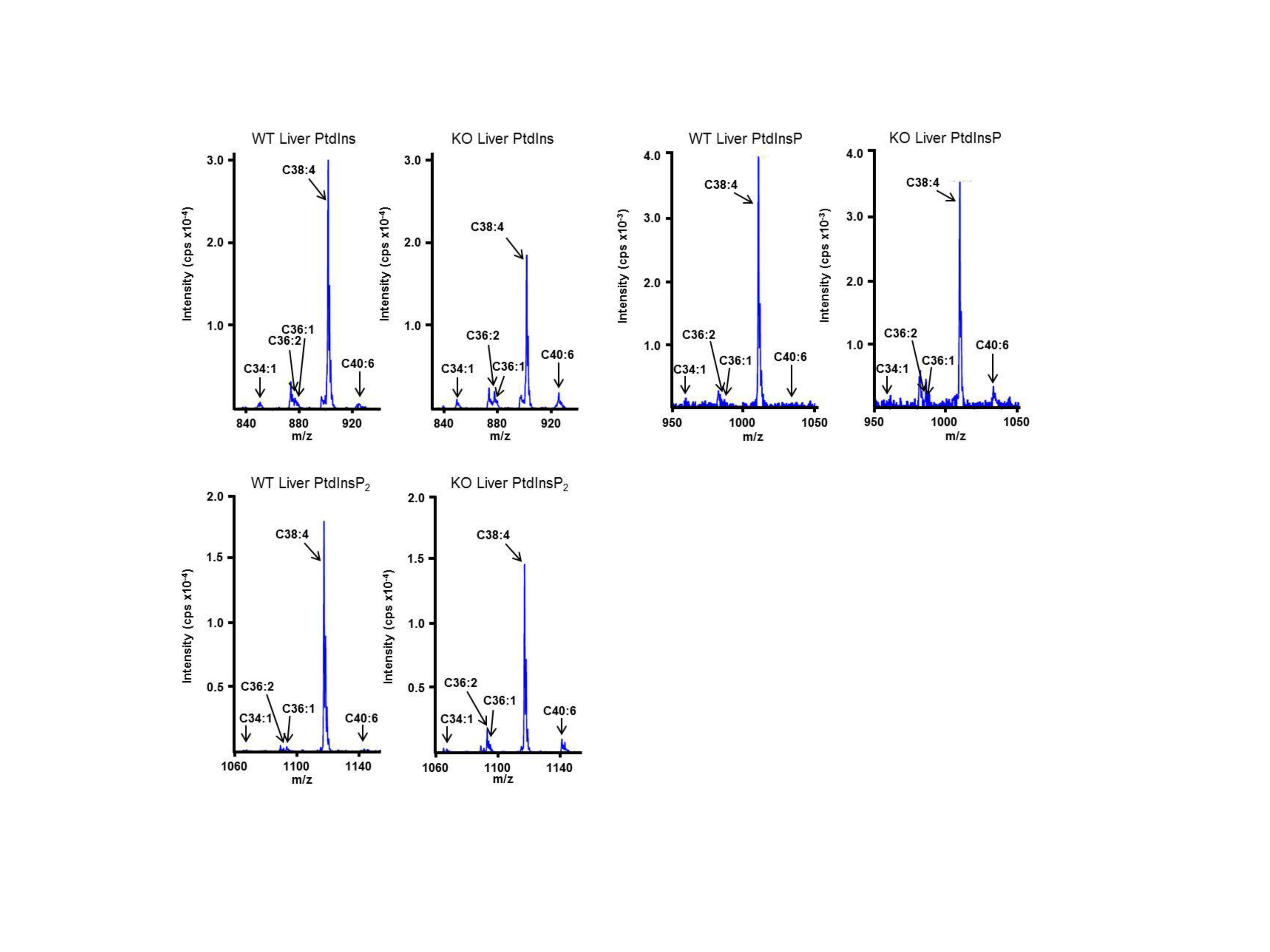

Supplement: Figure S3 — Neutral Loss Scans of Phosphoinositides from derivatized lipid extracts from LPIAT1+/+ and LPIAT1−/− liver tissue. Lipids were extracted from 5 mg of ground liver tissue from LPIAT1+/+ (WT) and LPIAT1−/− (KO) mice and analyzed by neutral loss on a QTRAP4000 mass spectrometer as described in Materials and Methods. Shown are neutral loss scans for phosphoinositides PtdIns, PtdInsP and PtdInsP2. Labeled are the five lipid molecular species from each scan that were targeted by MRM in subsequent analysis, giving fatty acids from the diacylglycerol unit. cps = counts per second. (TIF) [file pone.0058425.s003.tif]

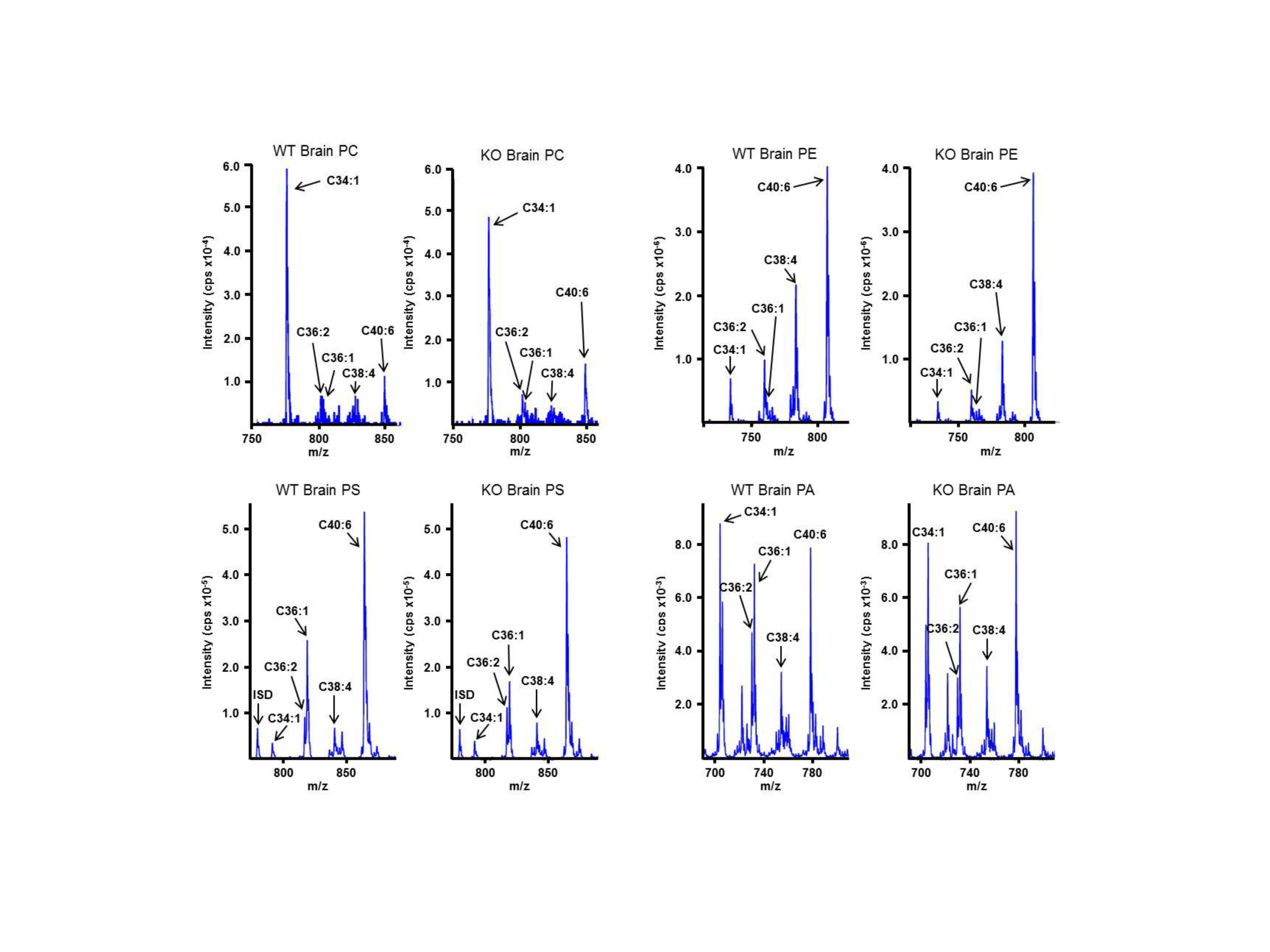

Supplement: Figure S4 — Neutral Loss Scans of Phospholipids from derivatized lipid extracts from LPIAT1+/+ and LPIAT1−/− brain tissue. Lipids were extracted from 5 mg of ground brain tissue from LPIAT1+/+ (WT) and LPIAT1−/− (KO) mice and analyzed by neutral loss on a QTRAP4000 mass spectrometer as described in Materials and Methods. Shown are neutral loss scans for PC, PE, PS and PA. Labeled are the five lipid molecular species from each scan that were targeted by MRM in subsequent analysis, giving fatty acids from the diacylglycerol unit. ISD = internal standard, cps = counts per second. (TIF) [file pone.0058425.s004.tif]

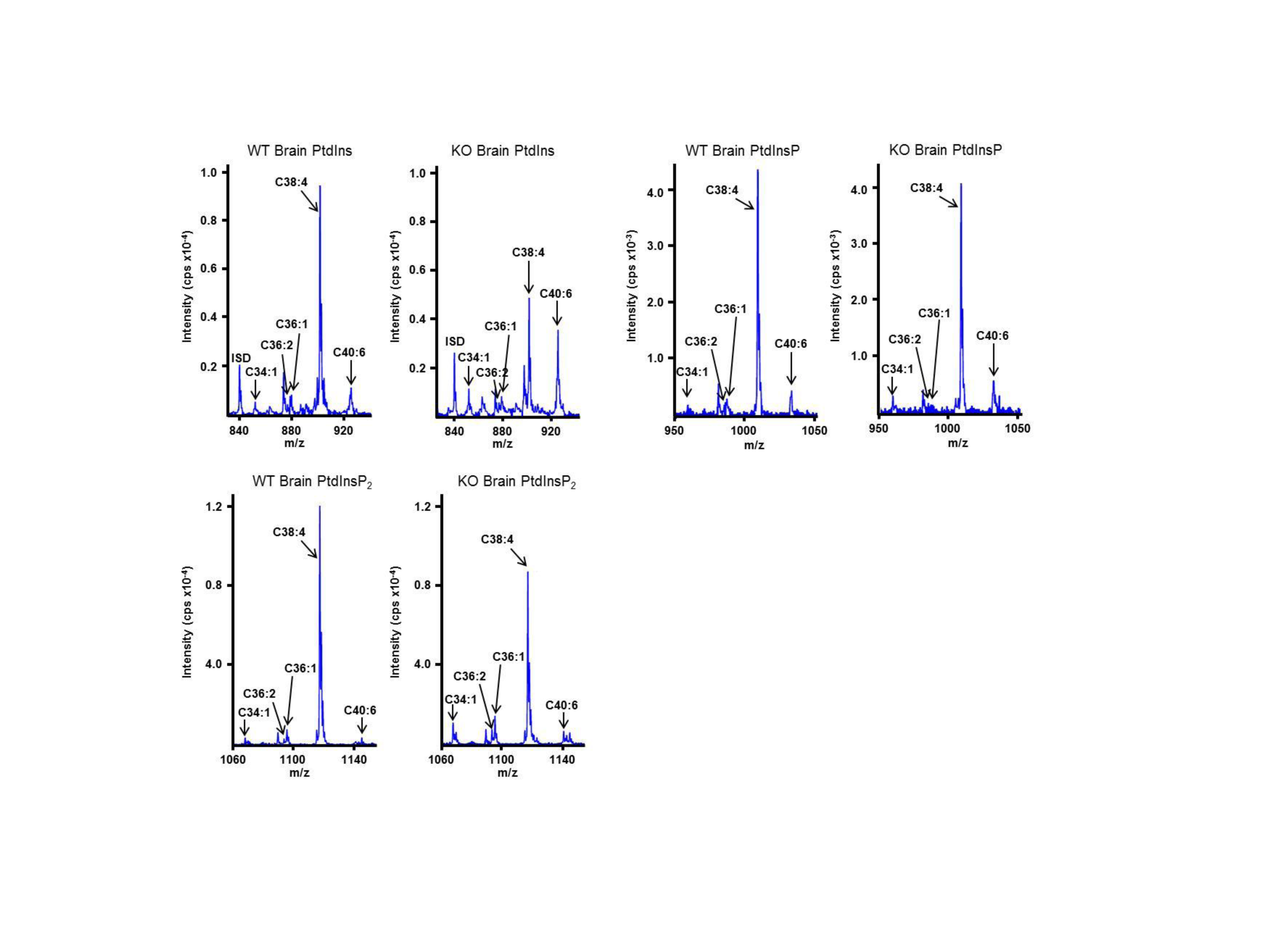

Supplement: Figure S5 — Neutral Loss Scans of Phosphoinositides from derivatized lipid extracts from LPIAT1+/+ and LPIAT1−/− brain tissue. Lipids were extracted from 5 mg of ground brain tissue from LPIAT1+/+ (WT) and LPIAT1−/− (KO) mice and analyzed by neutral loss on a QTRAP4000 mass spectrometer as described in Materials and Methods. Shown are neutral loss scans for phosphoinositides PtdIns, PtdInsP and PtdInsP2. Labeled are the five lipid molecular species from each scan that were targeted by MRM in subsequent analysis, giving fatty acids from the diacylglycerol unit. cps = counts per second. (TIF) [file pone.0058425.s005.tif]

A

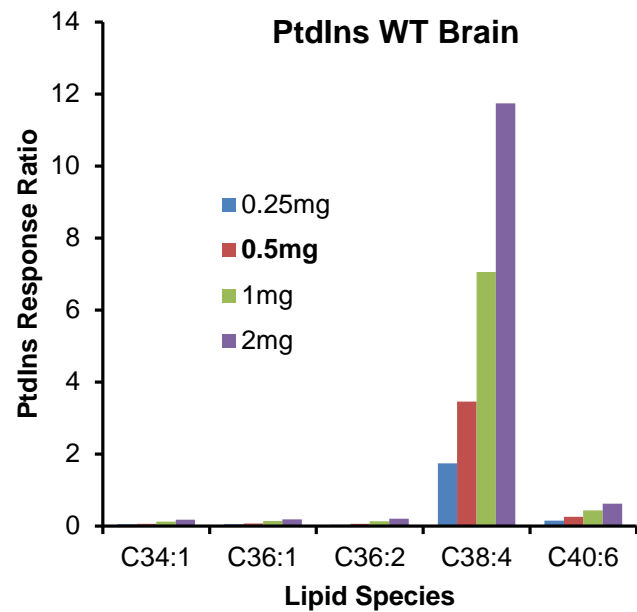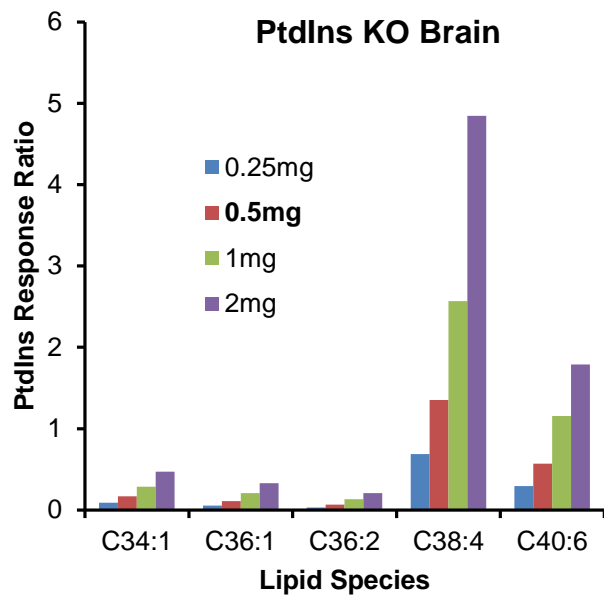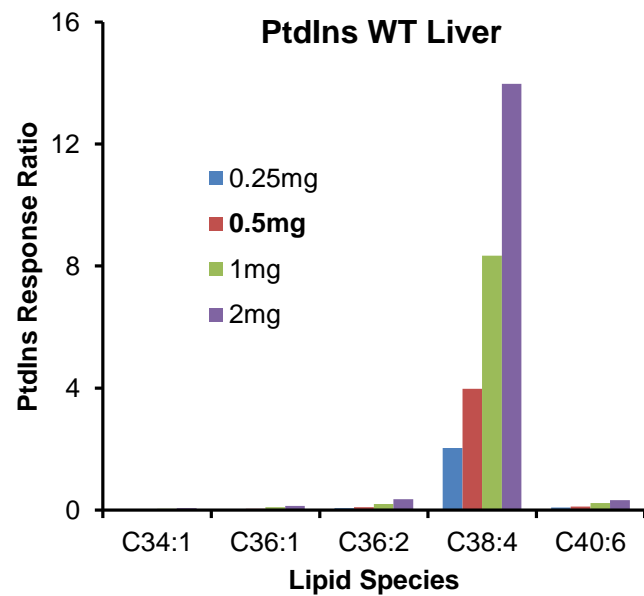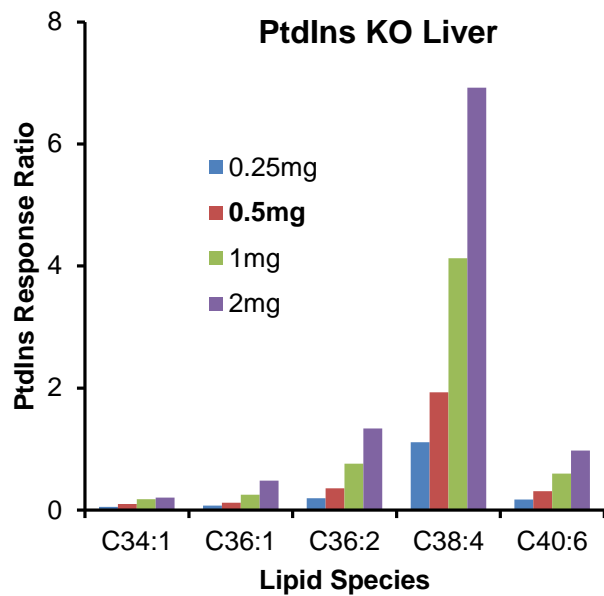

**B**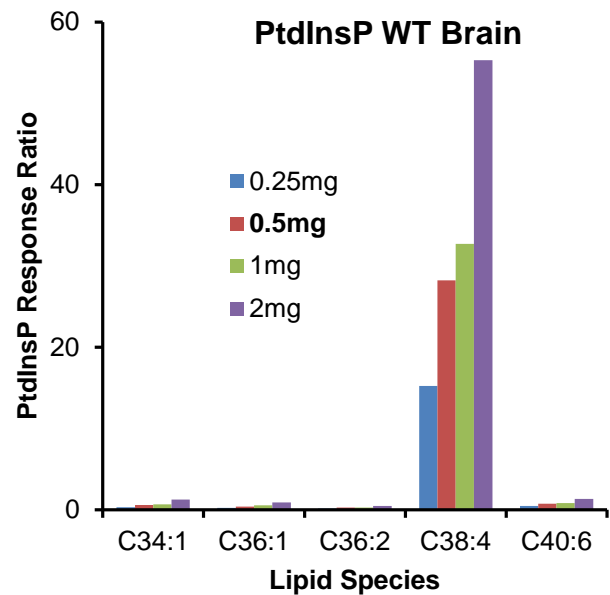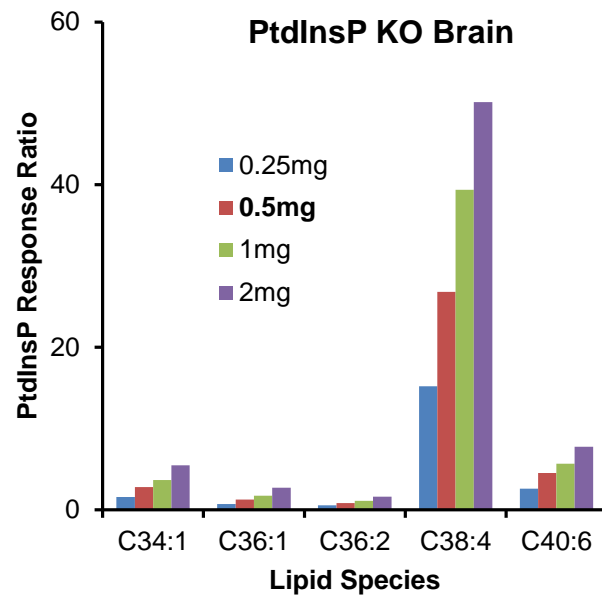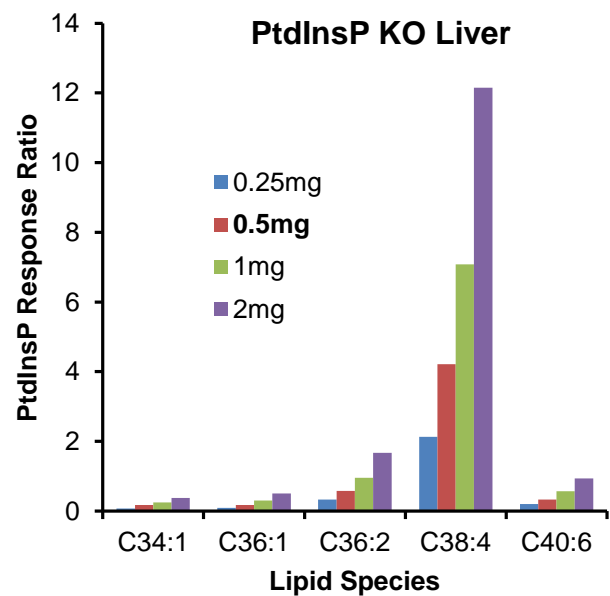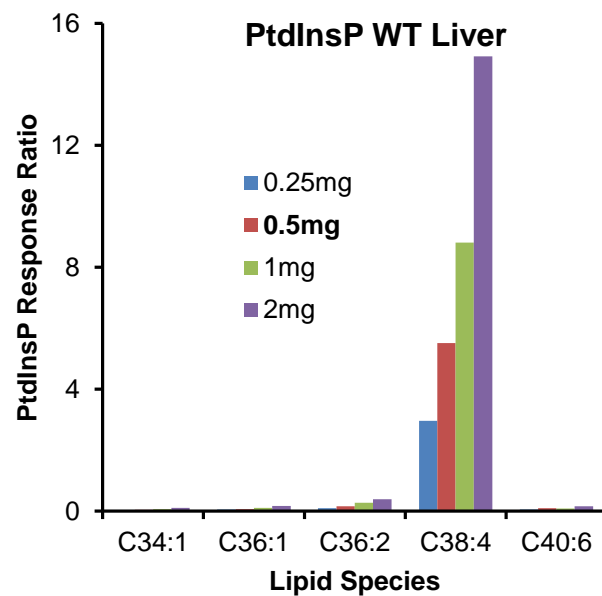

**C**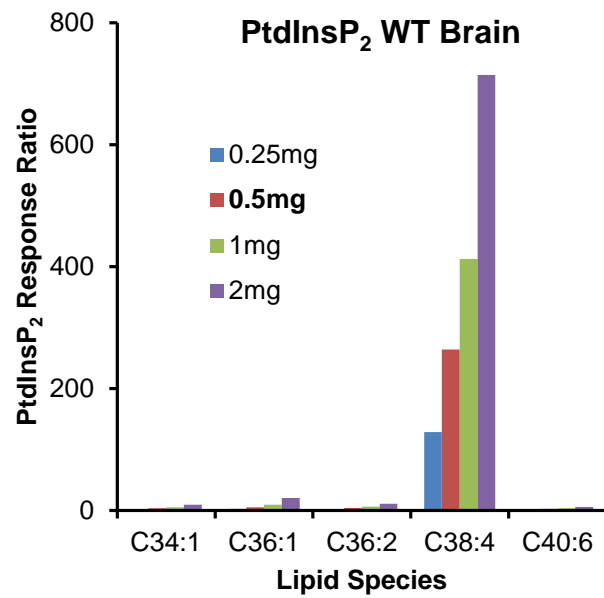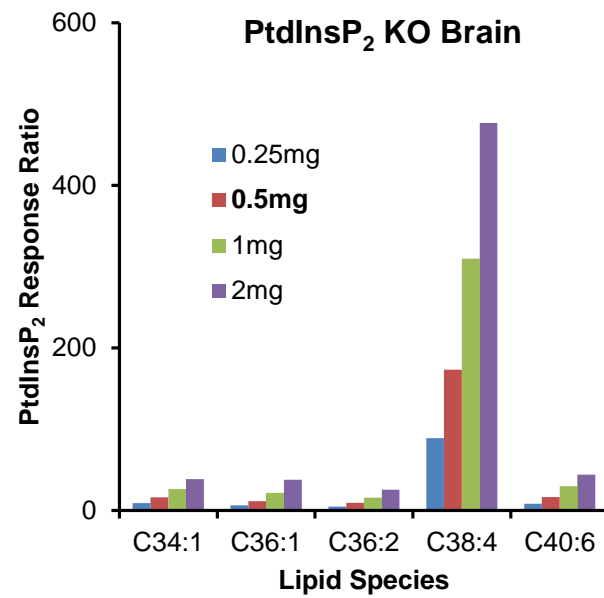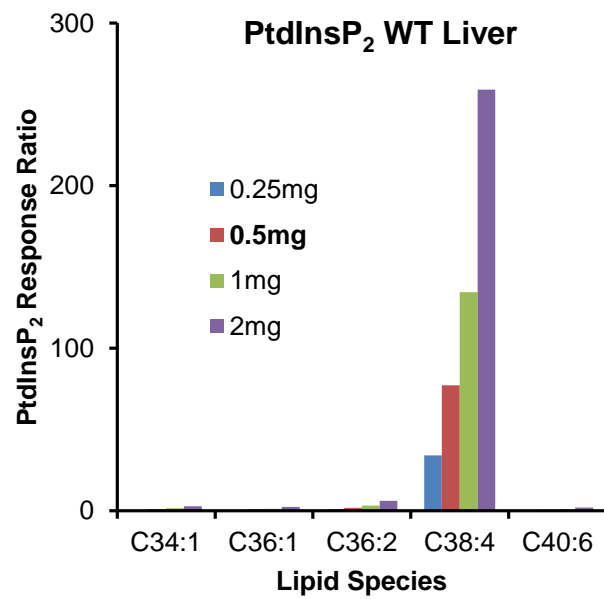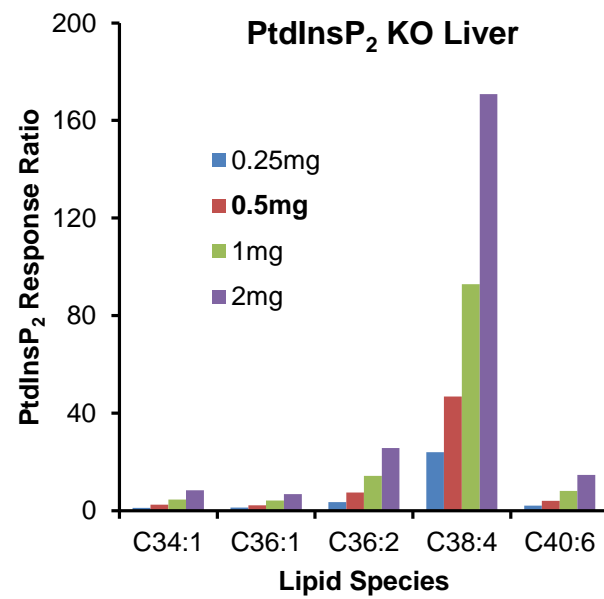

**D**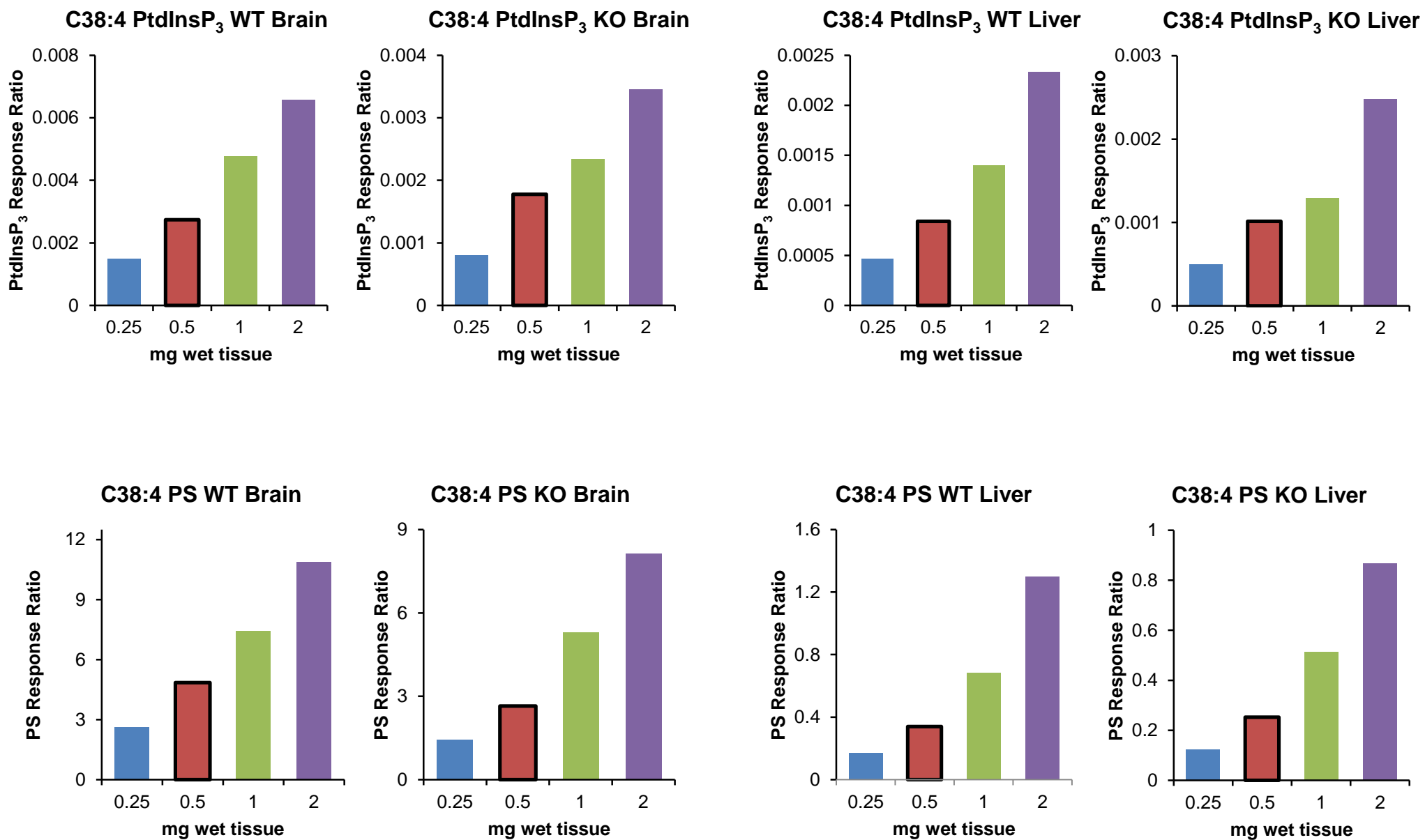

Supplement: Figure S6 — Linear measurement of phospholipid molecular species with increasing tissue amounts. Targeted molecular species of PtdIns (A), PtdInsP (B), PtdInsP2 (C) and C38∶4 PtdInsP3 and PS (D) were measured by mass spectrometry from increasing masses (0.25–2 mg tissue, independent dilutions, as indicated) from LPIAT1+/+ (WT) or LPIAT1−/− (KO) brain and liver samples, prepared as described in Materials and Methods. Data are presented as response ratios, with measurements normalized to relevant C16:C17-PtdIns, PtdInsP2 or PtdInsP3 added internal standards. PtdInsP values were normalized to C16:C17-PtdInsP2 standard. 0.5 mg samples (highlighted) were used for all subsequent analysis. (PDF) [file pone.0058425.s006.pdf]

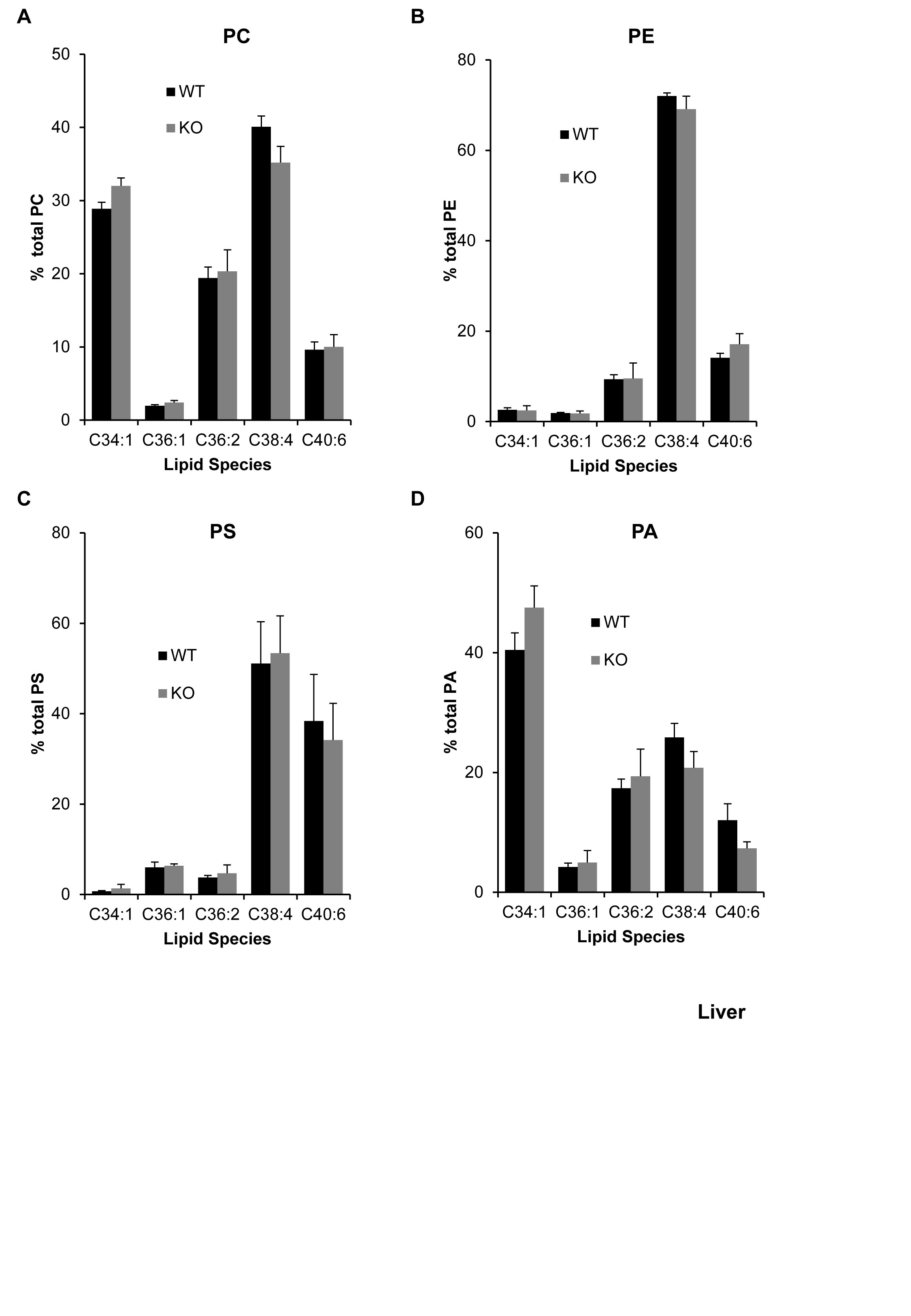

Supplement: Figure S7 — Effect of LPIAT1 knockout on relative amounts of phospholipid molecular species in the liver. Targeted molecular species of PC (A), PE (B), PS (C) and PA (D) from liver samples of mice expressing (LPIAT1+/+ (WT)) or lacking (LPIAT1−/− (KO)) LPIAT1 presented in Figure 2 were added to produce total levels of relevant lipids. Each molecular species was then calculated as a percentage of this total lipid value. Shown are mean ± SD, n = 4 for both WT and KO. Data were analyzed by T-test. (TIF) [file pone.0058425.s007.tif]

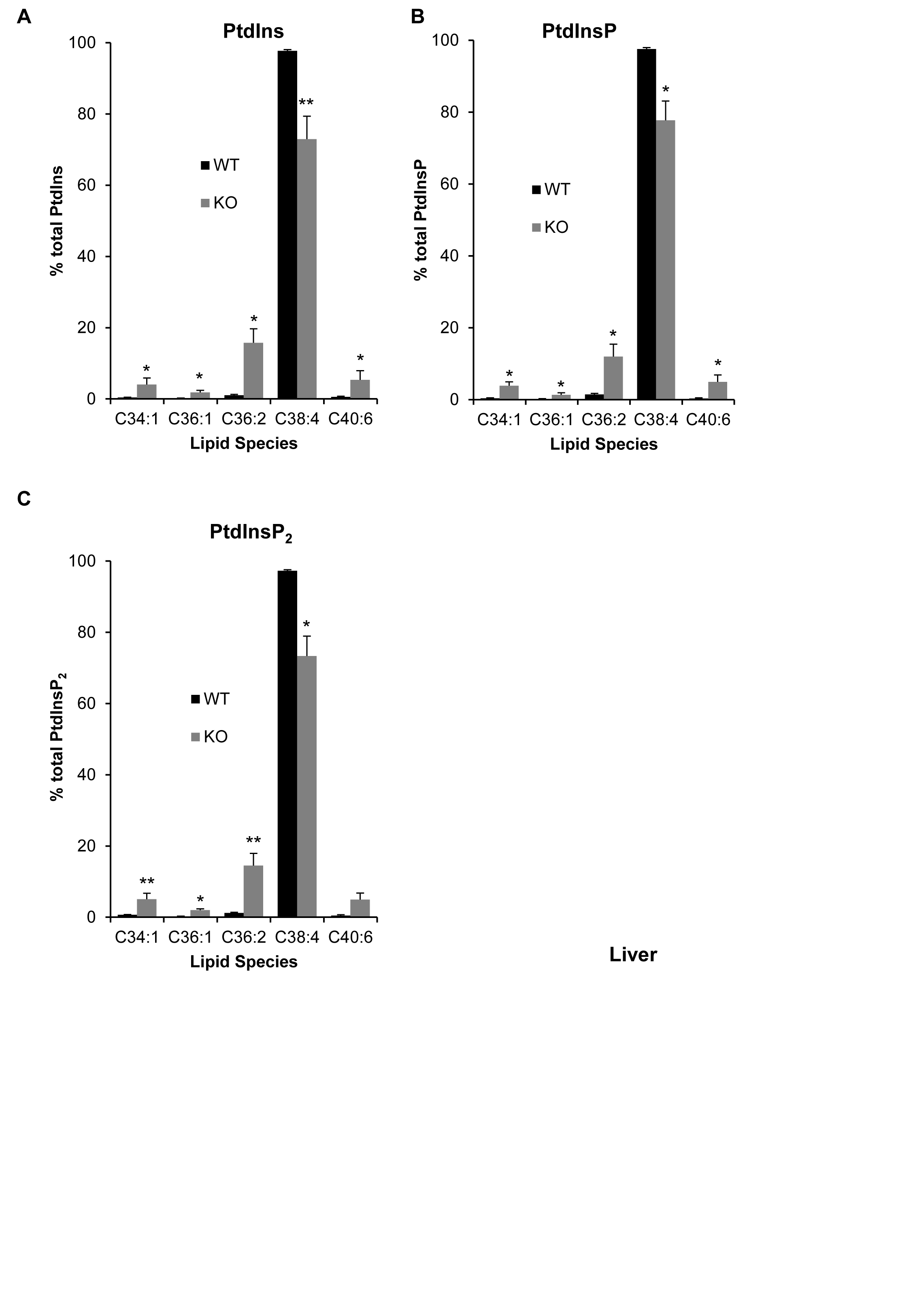

Supplement: Figure S8 — Effect of LPIAT1 knockout on relative amounts of phosphoinositide molecular species in the liver. Targeted molecular species of PtdIns (A), PtdInsP (B), and PtdInsP2 (C) from liver samples of WT or KO mice presented in Figure 3 were added to produce total levels of relevant lipids. Each molecular species was then calculated as a percentage of this total lipid value. Shown are mean ± SD, n = 4 for both WT and KO. Data were analyzed by T-test. (TIF) [file pone.0058425.s008.tif]

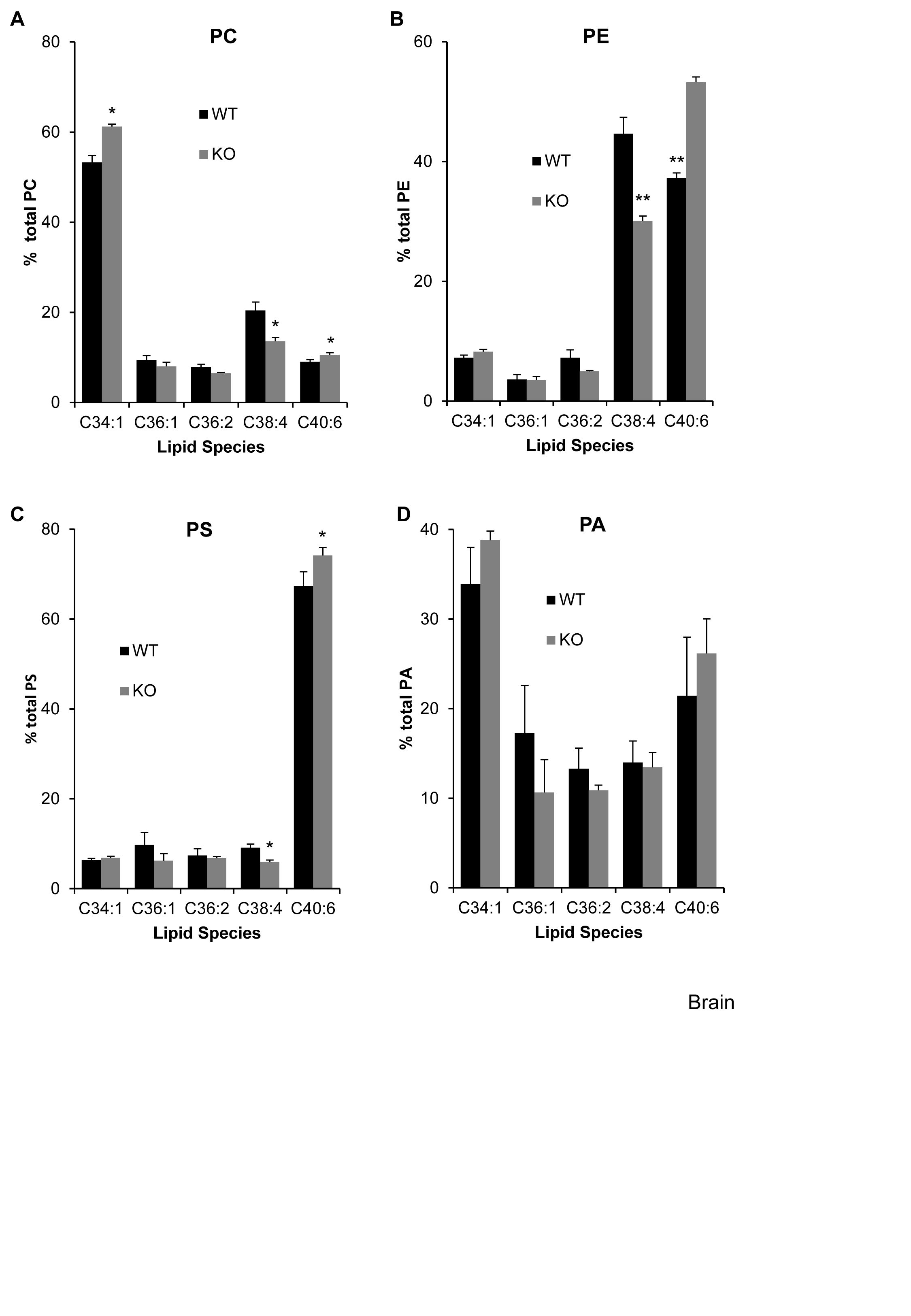

Supplement: Figure S9 — Effect of LPIAT1 knockout on relative amounts of phospholipid molecular species in the brain. Targeted molecular species of PC (A), PE (B), PS (C) and PA (D) from brain samples of mice expressing (LPIAT1+/+ (WT)) or lacking (LPIAT1−/− (KO)) LPIAT1 presented in Figure 5 were added to produce total levels of relevant lipids. Each molecular species was then calculated as a percentage of the total lipid pool. Shown are mean ± SD, n = 4 for both WT and KO. Data were analyzed by T-test. (TIF) [file pone.0058425.s009.tif]

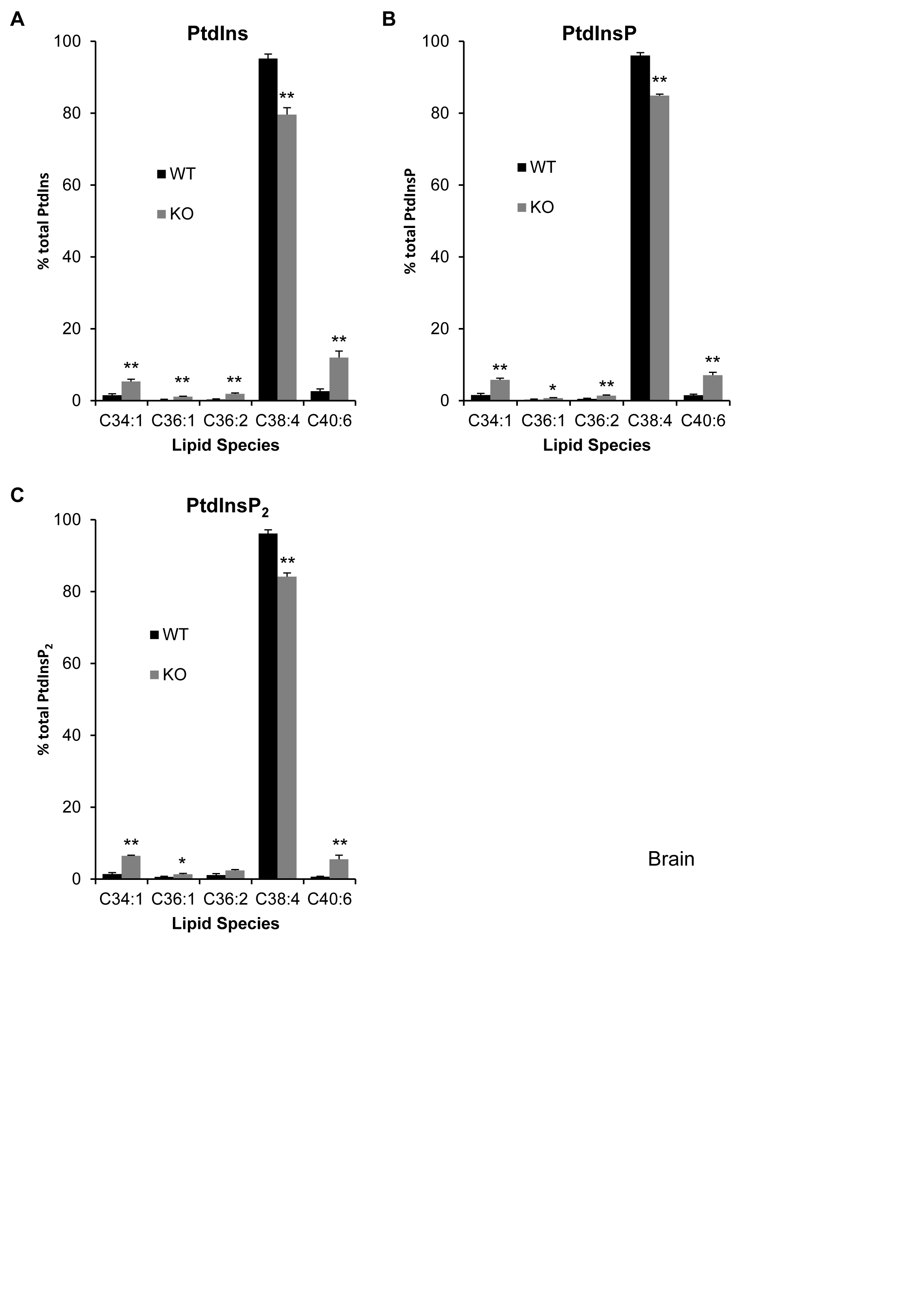

Supplement: Figure S10 — Effect of LPIAT1 knockout on relative amounts of phosphoinositide molecular species in the brain. Targeted molecular species of PtdIns (A), PtdInsP (B), and PtdInsP2 (C) from brain samples of WT or KO mice presented in Figure 6 were added to produce total levels of relevant lipids. Each molecular species was then calculated as a percentage of this total lipid value. Shown are mean ± SD, n = 4 for both WT and KO. Data were analyzed by T-test. (TIF) [file pone.0058425.s010.tif]
