# Supplementary material for: Lysophosphatidylinositol-Acyltransferase-1 (LPIAT1) Is Required to Maintain Physiological Levels of PtdIns and PtdInsP2 in the Mouse
Source: PLoS One. 2013 Mar 5;8(3):e58425. doi: 10.1371/journal.pone.0058425 (PMC3589398; doi:10.1371/journal.pone.0058425)
Supplement: Data S1 — MRM Transitions for Analysis of Internal Standards and Tissue Lipids on QTRAP4000 Mass Spectrometer. MRM transition parameters for detection of internal lipid standards and lipids in brain and liver tissue extracts, showing masses of derivatized head group (Q3- for neutral loss), and corresponding mass of parent lipid species (Q1). Parameters for each lipid were confirmed with synthetic standards. (PDF) [file pone.0058425.s011.pdf]

Internal Standards

Q1 Mass (Da)	Q3 Mass (Da)	
1163.519	565.519	**C17:0 C16:0 PtdIns(3,4,5)P_3 ISD**
1055.569	565.519	**C17:0 C16:0 PtdInsP_2 ISD**
839.559	565.519	**C17:0 C16:0 PtdIns ISD**
778.549	565.519	**C17:0 C16:0 PS ISD**
810.601	198.089	**C17:0 C20:4 PC ISD**
768.554	613.519	**C17:0 C20:4 PE ISD**
691.519	565.519	**C17:0 C20:4 PA ISD**
599.319	325.274	**C17:1-lyso PI ISD**
453.298	327.289	**C17:0-lyso PA ISD**

Tissue Lipids

Q1 Mass (Da)	Q3 Mass (Da)	
1225.535	627.535	**C38:4 PtdInsP_3**
1117.585	627.535	**C38:4 PtdInsP_2**
1009.575	627.535	**C38:4 PtdInsP**
901.575	627.535	**C38:4 PtdIns**
1093.585	603.535	**C36:2 PtdInsP_2**
985.575	603.535	**C36:2 PtdInsP**
877.575	603.535	**C36:2 PtdIns**
1095.601	605.551	**C36:1 PtdInsP_2**
987.591	605.551	**C36:1 PtdInsP**
879.591	605.551	**C36:1 PtdIns**
1067.585	577.535	**C34:1 PtdInsP_2**
959.575	577.535	**C34:1 PtdInsP**
851.575	577.535	**C34:1 PtdIns**
1141.585	651.535	**C40:6 PtdInsP_2**
1033.575	651.535	**C40:6 PtdInsP**
925.575	651.535	**C40:6 PtdIns**
825.625	198.089	**C38:4 PC**
782.565	627.535	**C38:4 PE**
840.565	627.535	**C38:4 PS**
753.535	627.535	**C38:4 PA**
801.625	198.089	**C36:2 PC**
758.565	603.535	**C36:2 PE**
816.565	603.535	**C36:2 PS**
729.535	603.535	**C36:2 PA**
803.641	198.089	**C36:1 PC**
760.581	605.551	**C36:1 PE**
818.581	605.551	**C36:1 PS**
731.551	605.551	**C36:1 PA**
775.625	198.089	**C34:1 PC**
732.565	577.535	**C34:1 PE**
790.565	577.535	**C34:1 PS**
703.535	577.535	**C34:1 PA**
849.625	198.089	**C40:6 PC**
806.565	651.535	**C34:1 PE**
864.565	651.535	**C34:1 PS**
777.535	651.535	**C34:1 PA**

554.335	341.305	**Lyso-PS**
496.335	341.305	**Lyso-PE**
539.395	198.089	**Lyso-PC**
467.305	341.305	**Lyso-PA**
615.345	341.305	**Lyso-PI**

Support Data D1. MRM Transitions for QTRAP Mass Spectrometer analysis of internal standards and tissue lipids.
